# Liver histology is associated with long-term clinical outcomes in patients with metabolic dysfunction–associated steatohepatitis

**DOI:** 10.1097/HC9.0000000000000423

**Published:** 2024-05-10

**Authors:** Zobair M. Younossi, Kamal Kant Mangla, Tina Landsvig Berentzen, Katrine Grau, Mette Skalshøi Kjær, Steen Ladelund, Louise Maymann Nitze, Crystal Coolbaugh, Chih-Yuan Hsu, Hannes Hagström

**Affiliations:** 1 Beatty Liver and Obesity Research Program, Inova Health System, Falls Church, Virginia, USA; 2 The Global NASH Council, Washington, District of Columbia, USA; 3 Novo Nordisk A/S, Søborg, Denmark; 4 Nashville Biosciences, Nashville, Tennessee, USA; 5 Department of Medicine, Huddinge, Karolinska Institutet, Stockholm, Sweden; 6 Division of Hepatology, Department of Upper GI Diseases, Karolinska University Hospital, Stockholm, Sweden

## Abstract

**Background:**

Few studies have examined the risk of long-term clinical outcomes in patients with metabolic dysfunction–associated steatohepatitis in relation to liver histology. We aimed to study this using a real-world cohort.

**Methods:**

Adults (N = 702) recorded on Vanderbilt University Medical Center’s Synthetic Derivative database (1984–2021) with evidence of metabolic dysfunction–associated steatohepatitis on liver biopsy were followed from the first biopsy until the first clinical event or last database entry (median: 4.7 y). Risks of cirrhosis (N = 650), other noncirrhotic liver-related (N = 702) and cardiovascular-related outcomes (N = 660), and mortality due to liver, cardiovascular, or cancer events (N = 660) were determined as a function of baseline histology (fibrosis stage [F], lobular inflammation grade [LI], hepatocyte ballooning grade [HB], and steatosis score) adjusting for sex, age, diabetes, and weight-loss surgery.

**Results:**

Cirrhosis risk was reduced for lower versus higher fibrosis stage (HR: F0–1 vs. F3: 0.22 [95% CI: 0.12–0.42]), LI1 versus LI2–3 (0.42 [0.19–0.97]), and HB1 versus HB2 (0.20 [0.08–0.50]). Lower fibrosis stage was associated with significantly lower risks of liver-related outcomes versus F4 cirrhosis (eg, F0–1: 0.12 [0.05–0.25]), whereas no differences were seen across baseline lobular inflammation, hepatocyte ballooning, and steatosis grades/scores. Lower versus higher lobular inflammation grade was associated with lower risks for liver-related outcomes in patients with weight-loss surgery. There was a trend for lower risks for cardiovascular-related and any long-term outcomes with lower versus higher fibrosis stage.

**Conclusions:**

Fibrosis stage and lobular inflammation and hepatocyte ballooning grades predict the risk of long-term outcomes, supporting the use of these histological features as potential surrogate markers of disease progression or clinical outcomes.

## Graphical abstract

**Figure:**
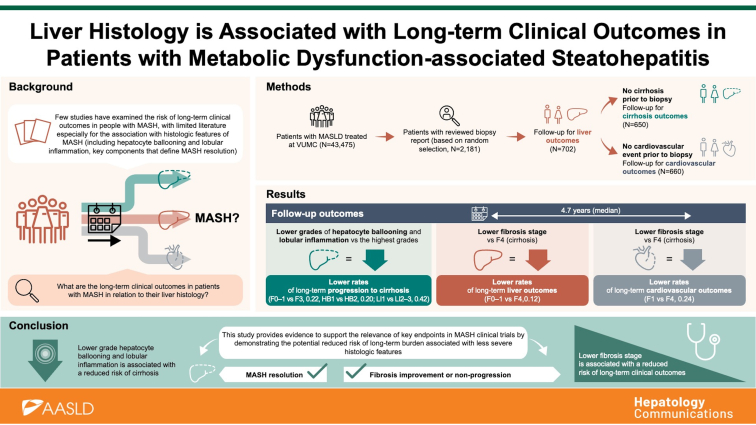

## INTRODUCTION

The prevalence of metabolic dysfunction–associated steatotic liver disease (MASLD) and its inflammatory subtype metabolic dysfunction–associated steatohepatitis (MASH), which is characterized by hepatic inflammation with hepatocellular ballooning and has the potential for progression to fibrosis, is increasing worldwide.^1–4^

Over a 2-year period, ~20% of the patients with MASH and bridging fibrosis can be expected to progress to cirrhosis, with ~10% then progressing from cirrhosis to hepatic decompensation over a similar time frame.^1,5^ Patients with MASLD in general, especially those with advanced fibrosis, are also at increased risk of HCC.^6,7^ Liver-related and overall mortality are increased in individuals with MASH versus the general population and compared with patients with MASLD without fibrosis,^2,8–11^ and MASH is the fastest growing contributor to HCC-related deaths.^12^ Nevertheless, the leading cause of death among patients with MASH and MASLD is cardiovascular disease.^4,13,14^ Despite the increasing morbidity and mortality burden, there are no specific pharmacological treatments approved for patients with MASH.^15^

The gold standard for MASH diagnosis is a liver biopsy. In the clinical trial setting, end-of-treatment biopsies are also performed to assess the efficacy of potential new treatments in reducing fibrosis and achieving resolution of MASH (grade 0–1 for lobular inflammation and 0 for hepatocyte ballooning).^16,17^ However, most clinical trials have a relatively short duration and so are unable to show any effect on MASH-related long-term outcomes. In real-world practice, achieving MASH resolution or improvement of fibrosis is uncommon due to a lack of approved pharmacotherapy and challenges with current management guidelines for MASH. In addition, it is difficult to observe these histology outcomes in the real world through liver biopsies because of the potential risk of complications and cost associated with liver biopsies, leading to their limited usage.^18,19^ The result of this is little direct evidence regarding the association between liver histology and the long-term clinical outcomes in patients with MASH, especially from real-world practice.

There are a limited number of studies that have investigated the link between histology and long-term outcomes in MASH; most studies and meta-analyses conducted to date have focused specifically on the association between fibrosis stage and subsequent risk of clinical outcomes, particularly mortality, among patients with MASLD or MASH.^2,8–10,20–22^ The importance of improving fibrosis and achieving resolution of steatohepatitis in relation to long-term outcomes remains limited.

Considering the possibilities offered by the analysis of real-world data, this observational cohort study examined long-term liver-related and cardiovascular-related outcomes by baseline histology, and the potential for reduced outcome risks associated with less severe histologic features among patients with MASH-like liver histology in US clinical practice.

## METHODS

### Study design

Real-world data from patients aged 18–89 years were collated from de-identified electronic medical records (EMRs) entered into the Vanderbilt University Medical Center’s Synthetic Derivative (SD) database (https://www.vumc.org/dbmi/synthetic-derivative) between June 1984 and June 2021. The database covers more than 3.5 million patients from states in the southeastern USA.

Patients with an MASLD or MASH diagnosis (previously known as NAFLD and NASH, respectively, as defined by International Classification of Diseases [ICD] Clinical Modification [CM] codes; see Supplemental Digital Content, http://links.lww.com/HC9/A868 and http://links.lww.com/HC9/A869) and a pathology report for a liver biopsy procedure recorded in the database were extracted at random. Patients were required to have ≥1 ICD-9-CM code (571.8) and/or ≥1 ICD-10-CM code (K76.0, K75.81) for NAFLD (MASLD) or NASH (MASH) in their EMR. Original biopsy slides were not available. A central board–certified pathologist (Steven Held, MD, Nashville Biosciences) reviewed the written biopsy reports to identify patients who could be categorized as “more likely MASH.” Full biopsy evaluation criteria are given in the Supplemental Digital Content, http://links.lww.com/HC9/A870. These criteria were designed to apply a stepwise decision tree to standardize the interpretation of real-world liver biopsy report data collected over 2 decades of clinical care. Patients were included in the cohort if their liver biopsy pathology report was categorized as “more likely MASH” and the fibrosis stage was documented. Fibrosis stage and the components of the nonalcoholic fatty liver disease activity score (NAS), that is, the sum of scores/grades for steatosis, lobular inflammation, and hepatocyte ballooning,^16^ were extracted (or derived in cases where the information was not explicitly documented) for each patient. The analyses were conducted based on the number of reports with completed reviews available. Records were excluded from the analyses and not considered for biopsy review if patients had evidence of ≥1 ICD-9 or ICD-10 CM code for relevant conditions (eg, chronic liver disease other than MASLD or alcohol abuse; the full list is described in the Supplemental Digital Content, http://links.lww.com/HC9/A868, http://links.lww.com/HC9/A869, and http://links.lww.com/HC9/A870) at any time point in the EMR (pre-liver biopsy or post-liver biopsy); were pregnant, breastfeeding, or had gestational diabetes within ±9 months of the index date; had no record of fibrosis staging; or had a history of HCC, hepatic decompensation events, or liver transplantation before biopsy. In addition, for cirrhosis outcomes, patients with cirrhosis events before or up to 30 days after the index date (biopsy date) were excluded.

Patients were followed from the date of their first liver biopsy with histologic evidence of MASH, as judged by a pathologist based on a central review of the pathology report (index date), until their first clinical outcome or, for patients without a clinical outcome, their last EMR entry date. Baseline liver histology was ascertained from the liver biopsy pathology report used to categorize the patient as “more likely MASH” (fibrosis stage: F0–1, F2, F3, F4; lobular inflammation grade: LI0, 1, 2–3; hepatocyte ballooning grade: HB0, 1, 2; steatosis score: S0–1, 2, 3; and NAS: 0–2, 3–4, 5–8). Patients with S0 were considered to be “more likely MASH” if the biopsy report mentioned “steatohepatitis,” or if the stepwise approach based on the clinical judgement of the central pathologist concluded that they had MASH. The same was true for HB0 and LI0, where parameters could be interpreted as minimum or not qualified for a higher grade, but patients could still be suspected to have MASH. Data on comorbidities were extracted on the basis of ≥1 ICD code for the specified condition occurring before or on the patient’s index date. Data for baseline demographics, vital signs, laboratory measures, and medications were extracted for the period ±90 days of the index event. In the case of laboratory findings with multiple values on the same day, the baseline value was defined as the mean of the available measurements.

### Analytical method

#### Definition of outcomes

The incidence of clinical outcomes (from the liver biopsy index date to the first outcome date) was determined and incidence rates (person-years [PY]) were calculated accordingly. Clinical outcomes were defined by ICD-9-CM and ICD-10-CM or current procedural terminology codes (full list provided in the Supplemental Digital Content, http://links.lww.com/HC9/A868, http://links.lww.com/HC9/A869, and http://links.lww.com/HC9/A870). Analyses were performed for the endpoints of progression to cirrhosis (for patients with F0–3 at baseline); composite and individual liver-related outcomes (any of the following: HCC, Model for End-Stage Liver Disease score ≥15, hepatic decompensation, liver transplant, or liver-related mortality); composite and individual cardiovascular-related outcomes (any of the following: heart failure, myocardial infarction, ischemic stroke, unstable angina, coronary revascularization, and cardiovascular-related mortality for patients with no cardiovascular outcome events at baseline); and any long-term outcome (any liver-related [excluding cirrhosis] and cardiovascular-related outcomes, and mortality due to liver, cardiovascular, or cancer events). The incidence of an individual event (except for mortality) within each composite outcome (liver-related and cardiovascular-related outcomes) was also analyzed. All-cause mortality was not included in the definition of any long-term outcome and mortality events were not analyzed individually due to scarcity of outcome events.

#### Main analytical approach

HRs and 95% CIs for clinical outcomes were derived as a function of baseline histology, using Fine–Gray and Cox modeling for nonfatal events and total outcome event, respectively, adjusted for sex, baseline age, diabetes, and weight-loss surgery status. All HRs were adjusted unless otherwise specified. Individual models were used for each histologic feature using the higher stages/grades/scores of fibrosis, lobular inflammation, hepatocyte ballooning, and steatosis as reference values to estimate the potential reduced risk of long-term outcomes associated with having less severe histologic features compared with more progressed features. The Aalen–Johansen method with death as a competing risk was used to estimate the cumulative probabilities of nonfatal events for each subtype of fibrosis stages and baseline liver-related histological parameters.

The main analysis was performed on available fibrosis stage data and scores/grades of steatosis, inflammation, and hepatocyte ballooning, with a sensitivity analysis using multiple imputations to account for missing values of NAS components (scores/grades of steatosis, inflammation, and hepatocyte ballooning). The association between NAS and time to outcomes was analyzed using multiple imputations because of a high number of missing values. Multiple imputations were performed for each missing component of the NAS score (with 5 imputations) through additive regression, bootstrapping, and predictive mean matching. The reported result was the average based on the 5 imputed data sets.

Missing values for time-to-outcome and censored status were not imputed and were excluded from the regression analysis. Mortality status at the end of follow-up was treated as censored rather than missing if no mortality occurred during follow-up.

All statistical analyses were performed using R version 4.0.3 and the R packages survival 3.2-7, survminer 0.4.9, xtable 1.8-4, and Hmisc 4.4.2.

Further detail on the statistical methodology is provided in the Supplemental Digital Content, http://links.lww.com/HC9/A870.

### Exploratory weight-loss surgery subgroup analysis

Weight-loss surgery status was determined by analysis of current procedural terminology codes. While not part of the original analysis, a high proportion of patients were found to have had weight-loss surgery in the study cohort. To explore the potential effect of this on the results, a post hoc subgroup analysis was performed, stratifying by weight-loss surgery status at baseline for all composite outcomes. Analyses were not conducted for individual outcome events due to the limited cohort sizes and outcome rates in subgroups. An additional exploration of the entire database (following additional accrual of patient data in the SD database up to August 31, 2021) was conducted to understand the overlap between weight-loss surgery and biopsy based on the observed proportion of patients with weight-loss surgery performed on the same day as biopsy. Cumulative probabilities for 1-, 3-, and 5-year risk were extracted from Aalen–Johansen curve estimates at the respective time points.

### Exploratory analysis of FIB-4 for liver fibrosis

An exploratory analysis was conducted to assess changes in Fibrosis-4 Index (FIB-4; biomarker of liver fibrosis) in the absence of liver biopsy. The association between time-to-outcome event and changes from baseline in FIB-4 values at 360 (±90) days was analyzed. Patients with FIB-4 information available at baseline (near the index date) and at 360 (±90) days from the index date were selected for the analysis. Patients were followed from 30 days after the second FIB-4 measurement. Patients with events for respective outcome analyses before the second measurement date for FIB-4 +30 days were excluded, as were patients who were lost to follow-up before 30 days past the date of the 360-day FIB-4 follow-up. Loss to follow-up was defined based on the latest EMR entry date.

## RESULTS

### Cohort selection

**FIGURE 1:**
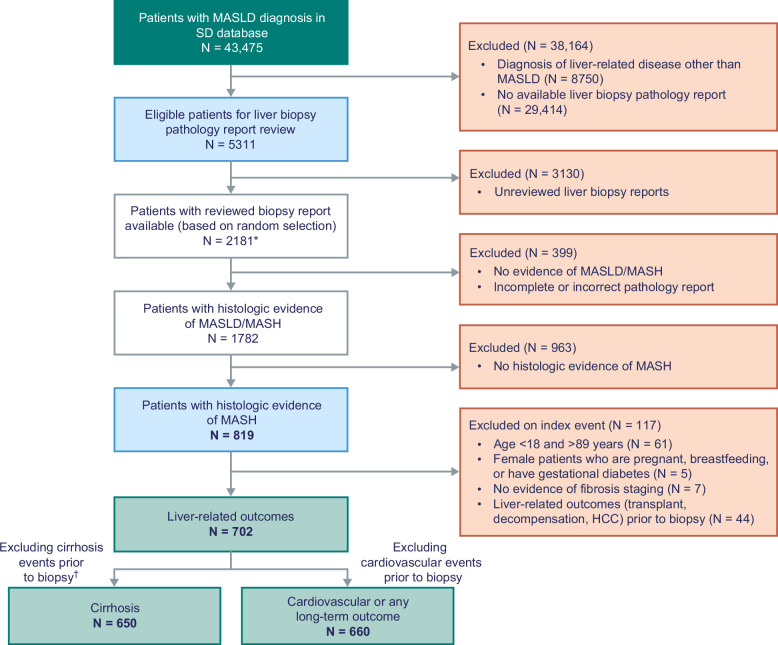
Cohort selection. *Analyses were conducted based on the number of reports with completed reviews available. ^†^Patients with baseline fibrosis classification F4 or those with a cirrhosis event before or up to 30 days after liver biopsy were excluded. Abbreviations: MASH, metabolic dysfunction–associated steatohepatitis; MASLD, metabolic dysfunction–associated steatotic liver disease; SD, Synthetic Derivative.

Among 43,475 patients in the SD database with a diagnosis of MASLD, 5311 had a liver biopsy pathology report available. A total of 2181 reviewed reports (based on random selection) were available at the time of study initiation, of which 399 were excluded because the report did not mention evidence of MASLD/MASH or was incomplete. Based on the central pathologist’s review, 819 patients were found to have histologic evidence of MASH-like pathology, but 117 of these patients met one or more exclusion criteria (Figure 1). A total of 702 patients with MASH-like histology were therefore included in the analyses of liver-related outcomes; 660 of these individuals—with no history of cardiovascular events—were included in the analysis of cardiovascular-related outcomes and any long-term outcome, and 650 patients (with no evidence of cirrhosis at baseline) were included in the analysis of cirrhosis outcomes.

### Description of patient characteristics and distribution by baseline liver histology

**FIGURE 2:**
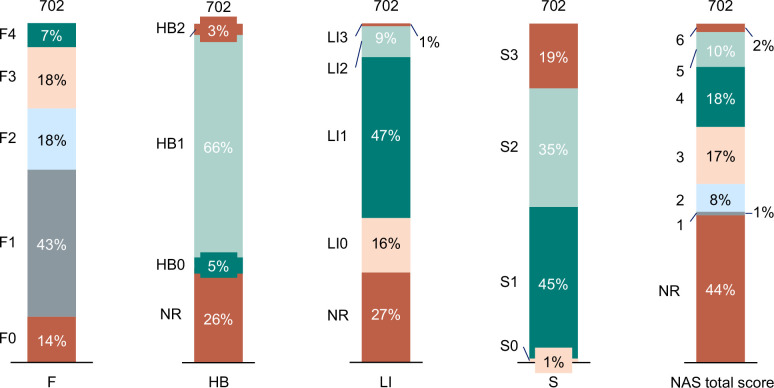
Baseline liver histology. NAS total scores were considered “unknown” if at least 1 subcomponent of the score (eg, hepatocyte ballooning, lobular inflammation, or steatosis) was not documented in the liver biopsy pathology report. Abbreviations: F, fibrosis stage; HB, hepatocyte ballooning grade; LI, lobular inflammation grade; NAS, nonalcoholic fatty liver disease activity score; NR, not reported; S, steatosis score.

Of the 702 patients included in the analysis of liver-related outcomes, all patients had fibrosis stage and steatosis score available at baseline, 515 (73%) had baseline data on lobular inflammation, and 517 (74%) had baseline data on hepatocyte ballooning (Table 1). A baseline NAS was available or could be derived for 56% of the patients. Most patients (57%) had liver fibrosis stage F0–1, 36% had stages F2 or F3, and 7% had stage F4 (Figure 2; see also distribution of other histopathologic components).

**TABLE 1:** Baseline characteristics by fibrosis stage for the analysis of liver-related outcomes

	N	F0–1 (N = 400)	F2 (N = 123)	F3 (N = 128)	F4 (N = 51)	Total (N = 702)
Demographics						
Female, %	702	72	64	67	67	70
Age, median (IQR), y	702	48 (40–56)	49 (44–57)	52 (44–60)	59 (51–64)	50 (42–58)
BMI, median (IQR), kg/m^2^	685	44 (38–50)	45 (38–51)	41 (35–48)	40 (35–46)	44 (37–50)
White, %	702	87	93	92	96	90
Hispanic, %	702	1	2	3	2	2
Smoker, %	702	31	37	38	35	33
Follow-up, median (IQR), y	702	5.1 (2.7–7.8)	4.8 (2.9–7.7)	4.3 (3.0–6.5)	4.0 (2.5–5.5)	4.7 (2.8–7.6)
Medical history, %						
Obesity	702	82	82	78	73	80
Hypertension	702	74	74	62	73	72
Dyslipidaemia	702	56	54	45	63	54
Type 2 diabetes	702	44	54	62	76	51
Prior weight-loss surgery	702	74	77	59	29	68
Clinical and laboratory data, median (IQR)						
ALT, U/L	552	36 (25–60)	39 (26–62)	47 (25–83)	48 (28–101)	39 (26–70)
AST, U/L	553	32 (23–52)	30 (24–48)	38 (23–60)	38 (26–68)	33 (23–54)
ALP, U/L	553	81 (67–100)	81 (66–94)	81 (68–95)	87 (71–98)	81 (67–97)
HbA_1c_, %	287	6.5 (5.8–7.7)	6.4 (5.9–7.7)	6.7 (5.8–7.5)	6.7 (5.4–7.1)	6.5 (5.8–7.6)
HDL cholesterol, mg/dL	152	41 (32–48)	42 (35–46)	44 (36–49)	46 (40–48)	42 (34–48)
LDL cholesterol, mg/dL	151	92 (65–121)	105 (86–131)	108 (79–124)	108 (76–134)	103 (70–126)
Triglycerides, mg/dL	157	180 (106–270)	185 (126–280)	214 (144–252)	166 (125–228)	180 (118–261)
Biomarkers, median (IQR)						
FIB-4	501	0.90 (0.64–1.27)	1.11 (0.86–1.57)	1.40 (0.89–2.01)	2.65 (1.57–3.39)	1.07 (0.76–1.62)
NFS	455	-0.90 (-1.96–0.05)	-0.20 (-1.29–0.93)	-0.16 (-1.05–0.87)	1.25 (0.41–1.98)	-0.47 (-1.53–0.71)
APRI	501	0.29 (0.21–0.42)	0.37 (0.24–0.60)	0.45 (0.28–0.75)	0.78 (0.50–1.17)	0.35 (0.23–0.58)
AAR	501	0.82 (0.65–1.00)	0.85 (0.70–1.07)	0.94 (0.73–1.13)	1.07 (0.81–1.24)	0.87 (0.69–1.07)

*Note*: Type 2 diabetes status was assessed using an algorithm approach based on ICD coding and antidiabetic medications. Other medical history conditions were evaluated using ICD or CPT (for weight-loss surgery) coding. Smoking status was assessed as “ever,” “never,” or not reported.

Abbreviations: AAR, aspartate aminotransferase to alanine aminotransferase ratio; ALP, alkaline phosphatase; ALT, alanine aminotransferase; APRI, aspartate aminotransferase to platelet ratio index; AST, aspartate aminotransferase; BMI, body mass index; CPT, Current Procedural Terminology; F, fibrosis; FIB-4, Fibrosis-4 Index; HbA_1c_, glycated hemoglobin; ICD, International Classification of Diseases; NFS, nonalcoholic fatty liver disease fibrosis score.

Patient baseline data are shown by fibrosis stage in Table 1 and Supplemental Table S1, http://links.lww.com/HC9/A870, and by hepatocyte ballooning and lobular inflammation in Supplemental Tables S2 and S3, http://links.lww.com/HC9/A870. At index, patients (70% of whom were female) had a median (IQR) age of 50 (42–58) years and a median (IQR) body mass index of 44 (37–50) kg/m^2^ (Table 1); 68% (480/702) of patients had undergone weight-loss surgery at or before the index date. Patients with lower stages of fibrosis and grades of hepatocyte ballooning and lobular inflammation tended to be younger with higher body mass index and were less likely to have diabetes and more likely to have undergone weight-loss surgery than those with higher levels of these parameters (Table 1; Supplemental Tables S1–S3, http://links.lww.com/HC9/A870). No clear pattern was evident between baseline characteristics and degree of steatosis (Supplemental Table S4, http://links.lww.com/HC9/A870).

Of the 480 patients with baseline weight-loss surgery, 473 (98.5%) had their biopsy on the same day as surgery. Additional exploratory analysis covering all patients with MASLD/MASH without other liver-related comorbidities in the entire SD database suggested that only 10% (3627) of the 35,543 individuals with a diagnosis of MASLD/MASH had undergone weight-loss surgery. This proportion rose to 54% in patients who had a biopsy but was only 2% in patients without a biopsy.

### Analysis of long-term outcomes by fibrosis stage

**FIGURE 3:**
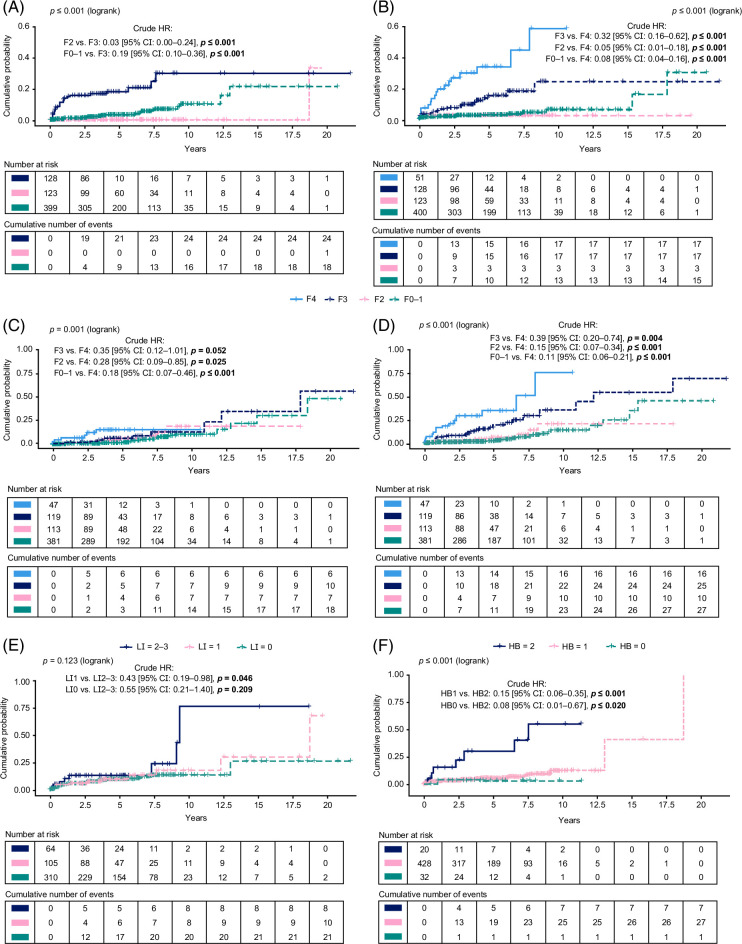
Cumulative probability plots for (A) cirrhosis, (B) liver-related outcomes, (C) cardiovascular-related outcomes, and (D) any long-term outcome by baseline fibrosis stage and for cirrhosis by (E) lobular inflammation and by (F) hepatocyte ballooning grades. Abbreviations: F, fibrosis stage; HB, hepatocyte ballooning grade; LI, lobular inflammation grade.

**FIGURE 4:**
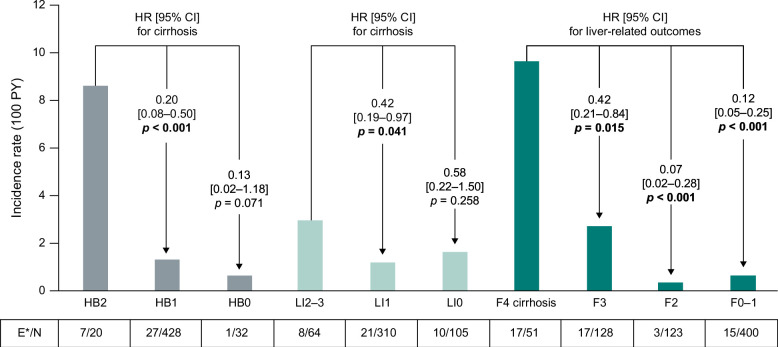
Risk of cirrhosis stratified by baseline lobular inflammation and hepatocyte ballooning grades, and risk of liver-related outcomes stratified by baseline fibrosis stage. Results for steatosis were inconclusive. *Outcomes (events) are shown as cirrhosis by hepatocyte ballooning and lobular inflammation grade, and as liver-related complications (includes HCC, Model for End-Stage Liver Disease score ≥15, and hepatic decompensation events) by fibrosis stage. HRs with 95% CIs adjusted for sex, baseline age, diabetes, and weight-loss surgery were calculated from Fine–Gray competing risk models. The model accounted for the competing risk of death. Significant *p* values are in bold. Abbreviations: E/N, events/total patients; F, fibrosis stage; HB, hepatocyte ballooning grade; LI, lobular inflammation grade; PY, person-years.

Cumulative probability plots are shown in Figure 3. Lower fibrosis stages were associated with lower rates of incident cirrhosis (Supplemental Table S5, http://links.lww.com/HC9/A870). The rate of incident cirrhosis per 100 PY for patients with F0–1 (18 events in 399 patients) versus F3 (24 events in 128 patients) was 0.84 versus 4.37 (HR [95% CI]: 0.22 [0.12–0.42], *p*<0.001), respectively. Lower fibrosis stages were also associated with significantly lower risks for liver-related outcomes (HCC, Model for End-Stage Liver Disease score ≥15, hepatic decompensation, and liver transplant): 0.69 per 100 PY for patients with F0–1 (15 events in 400 patients) versus 9.73 for those with F4 (17 events in 51 patients; HR [95% CI]: 0.12 [0.05–0.25], *p*≤0.001). HRs were also significant compared with F4 for patients with F2 (3 events in 123 patients; HR [95% CI]: 0.07 [0.02–0.28], *p*≤0.001) and F3 (17 events in 128 patients; HR [95% CI]: 0.42 [0.21–0.84], *p* = 0.015) (Figure 4). A significant association between lower fibrosis stages and reduced outcome risks was also identified individually for decompensation (Supplemental Table S5, http://links.lww.com/HC9/A870).

**FIGURE 5:**
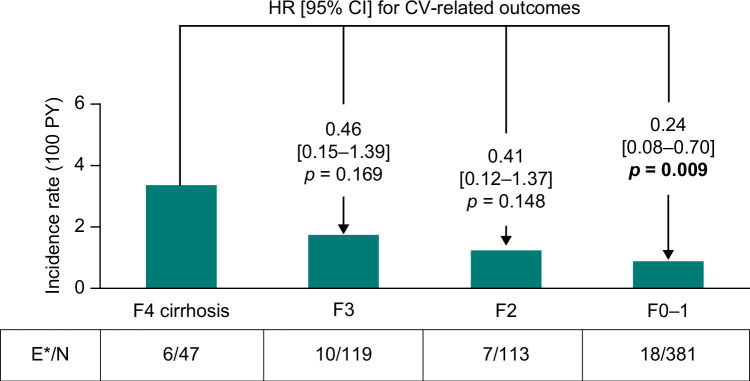
Risk of any cardiovascular event by fibrosis stage. *Outcomes (events) are shown as cardiovascular outcomes (heart failure, myocardial infarction, ischemic stroke, unstable angina, and coronary revascularization for patients with no cardiovascular outcome events at baseline) by fibrosis stage. HRs and 95% CIs were adjusted for sex, baseline age, diabetes, and weight-loss surgery, and were calculated from Fine–Gray competing risk models accounting for the competing risk of death. Significant *p* values are in bold. Abbreviations: E/N, events/total patients; F, fibrosis stage; PY, person-years.

There was a pattern of lower fibrosis stages being associated with a reduced risk for cardiovascular outcomes. This was significant for F0–1 (18 events in 381 patients) versus F4 (6 events in 47 patients) (HR [95% CI]: 0.24 [0.08–0.70], *p* = 0.009) (Figure 5). Significant associations were also identified for individual cardiovascular outcomes, specifically heart failure, myocardial infarction, and coronary revascularization, albeit with very low event rates (Supplemental Table S5, http://links.lww.com/HC9/A870).

Lower fibrosis stages were associated with a lower risk of any long-term outcome (liver-related or cardiovascular-related, or mortality due to liver, cardiovascular, or cancer events): 1.36 per 100 PY for patients with stage F0–1 (27 events in 381 patients) and 1.81 per 100 PY for F2 (10 events in 113 patients) versus 10.69 for those with stage F4 (16 events in 47 patients; HR [95% CI]: 0.16 [0.08–0.33], *p*<0.001 and HR [95% CI]: 0.23 [0.10–0.54], *p*<0.001, respectively; Supplemental Table S5, http://links.lww.com/HC9/A870).

The associations between lower (vs. higher) fibrosis stages and lower HRs for liver-related outcomes, including cirrhosis, remained mostly significant in the subgroup analysis by weight-loss surgery status (Tables 2 and 3). In both groups (with and without weight-loss surgery), patients with higher fibrosis stages were more likely to have a higher incidence of cardiovascular events versus lower fibrosis stages, although most comparisons did not reach statistical significance. Patients without weight-loss surgery generally had numerically higher incidence rates (per 100 PY) for long-term liver-related and cardiovascular outcomes than patients with weight-loss surgery.

**TABLE 2:** HRs of long-term liver-related outcomes, cirrhosis, and cardiovascular events stratified by baseline fibrosis stage and lobular inflammation grade in patients without weight-loss surgery

No weight-loss surgery								
Outcome	Histologic characteristic	N	Events	Incidence rate per 100 PY	HR [95% CI] *p*	1-year cumulative probability [95% CI]	3-year cumulative probability [95% CI]	5-year cumulative probability [95% CI]
Any liver-related outcomes	Fibrosis stage							
	F4	36	14	11.58	Reference	0.17 [0.04–0.28]	0.33 [0.15–0.48]	0.39 [0.17–0.54]
	F3	53	11	4.47	0.43 [0.18–1.00] ***p*** **=0.049**	0.04 [0–0.09]	0.13 [0.03–0.22]	0.26 [0.1–0.39]
	F2	28	2	1.03	0.08 [0.02–0.40] ***p*** **=0.002**	0.07 [0–0.16]	0.07 [0–0.16]	0.07 [0–0.16]
	F0–1	105	4	0.56	0.05 [0.01–0.17] ***p*** **<0.001**	0.01 [0–0.03]	0.01 [0–0.03]	0.01 [0–0.03]
	Lobular inflammation grade							
	LI2–3	26	3	3.16	Reference	0.08 [0–0.18]	0.12 [0–0.24]	0.12 [0–0.24]
	LI1	112	14	2.10	0.98 [0.28–3.52] *p* **=**0.98	0.06 [0.01–0.1]	0.09 [0.03–0.14]	0.11 [0.05–0.17]
	LI0	26	4	2.17	0.95 [0.20–4.43] *p* **=**0.95	0.04 [0–0.11]	0.13 [0–0.27]	0.13 [0–0.27]
Cirrhosis	Fibrosis stage							
	F3	53	13	5.48	Reference	0.16 [0.05–0.26]	0.19 [0.07–0.29]	0.22 [0.08–0.33]
	F2	28	1	0.51	0.09 [0.01–0.71] ***p*** **=0.022**	0 [0–0]	0 [0–0]	0 [0–0]
	F0–1	104	10	1.51	0.27 [0.11–0.63] ***p*** **=0.002**	0.01 [0–0.03]	0.02 [0–0.05]	0.05 [0–0.1]
	Lobular inflammation grade							
	LI2–3	21	3	4.36	Reference	0.05 [0–0.15]	0.05 [0–0.15]	0.05 [0–0.15]
	LI1	98	14	2.40	0.64 [0.18–2.32] *p* **=**0.50	0.07 [0.01–0.12]	0.09 [0.03–0.15]	0.14 [0.06–0.21]
	LI0	21	5	2.87	0.69 [0.15–3.10] *p* **=**0.62	0.05 [0–0.13]	0.05 [0–0.13]	0.05 [0–0.13]
Cardiovascular events	Fibrosis stage							
	F4	33	5	3.92	Reference	0.06 [0–0.14]	0.14 [0–0.25]	0.18 [0.02–0.31]
	F3	49	3	1.25	0.20 [0.04–1.03] *p* **=**0.055	0 [0–0]	0 [0–0]	0 [0–0]
	F2	25	3	2.12	0.43 [0.07–2.59] *p* **=**0.354	0 [0–0]	0.05 [0–0.14]	0.05 [0–0.14]
	F0–1	94	8	1.30	0.19 [0.04–0.94] ***p*** **=0.042**	0 [0–0]	0.01 [0–0.04]	0.01 [0–0.04]
	Lobular inflammation grade							
	LI2–3	23	2	2.32	Reference	0 [0–0]	0.14 [0–0.31]	0.14 [0–0.31]
	LI1	103	10	1.64	0.47 [0.10–2.35] *p* **=**0.359	0.01 [0–0.03]	0.02 [0–0.05]	0.04 [0–0.08]
	LI0	23	4	2.94	0.83 [0.14–4.84] *p* **=**0.836	0 [0–0]	0.06 [0–0.16]	0.06 [0–0.16]
Any long-term outcome	Fibrosis stage							
	F4	33	13	12.26	Reference	0.18 [0.04–0.31]	0.33 [0.14–0.48]	0.4 [0.16–0.57]
	F3	49	12	5.50	0.45 [0.19–1.03] *p*=0.059	0.04 [0–0.1]	0.12 [0.01–0.21]	0.23 [0.07–0.36]
	F2	25	5	3.58	0.29 [0.09–0.93] ***p*** **=0.037**	0.08 [0–0.18]	0.13 [0–0.25]	0.13 [0–0.25]
	F0–1	94	9	1.47	0.09 [0.03–0.25] ***p*** **<0.001**	0.01 [0–0.03]	0.03 [0–0.06]	0.03 [0–0.06]
	Lobular inflammation grade							
	LI2–3	23	4	4.72	Reference	0.04 [0–0.12]	0.23 [0–0.4]	0.23 [0–0.4]
	LI1	103	18	3.03	0.74 [0.24–2.27] *p* **=**0.602	0.06 [0.01–0.11]	0.1 [0.03–0.16]	0.11 [0.04–0.17]
	LI0	23	7	5.86	1.17 [0.33–4.11] *p* **=**0.805	0.04 [0–0.12]	0.16 [0–0.3]	0.16 [0–0.3]

*Note*: Significant *p* values are in bold. HRs were adjusted for sex, baseline age, and diabetes status. Association with hepatocyte ballooning was analyzed, but results were inconclusive, albeit with a significantly reduced risk of cirrhosis observed with HB1 versus HB2 in patients without weight-loss surgery. Cumulative probabilities for 1-, 3-, and 5-year risk were extracted from Aalen–Johansen curve estimates at the respective time points. Liver-related outcomes were defined as any of the following: HCC, MELD ≥15, hepatic decompensation, liver transplant, or liver-related mortality. Cirrhosis was analyzed separately in patients with F0–3 at baseline and not included in liver-related outcomes. Cardiovascular-related outcomes included any of the following: heart failure, myocardial infarction, ischemic stroke, unstable angina, coronary revascularization, and cardiovascular-related mortality for patients with no cardiovascular outcome events at baseline. Any long-term outcome was defined as any liver-related (excluding cirrhosis) and cardiovascular-related outcomes, and mortality due to liver, cardiovascular, or cancer events.

Abbreviations: F, fibrosis stage; HB, hepatocyte ballooning grade; LI, lobular inflammation grade; MELD, Model for End-Stage Liver Disease score; PY, person-years.

**TABLE 3:** HRs of long-term liver-related outcomes, cirrhosis, and cardiovascular events stratified by baseline fibrosis stage and lobular inflammation grade in patients with weight-loss surgery

Weight-loss surgery								
Outcome	Histologic characteristic	N	Events	Incidence rate per 100 PY	HR [95% CI] *p*	1-year cumulative probability [95% CI]	3-year cumulative probability [95% CI]	5-year cumulative probability [95% CI]
Any liver-related outcomes	Fibrosis stage							
	F4	15	3	5.58	Reference	0.07 [0–0.18]	0.22 [0–0.41]	0.22 [0–0.41]
	F3	75	6	1.64	0.48 [0.12–2.01] *p* **=**0.32	0.05 [0–0.1]	0.05 [0–0.1]	0.09 [0–0.17]
	F2	95	1	0.20	0.06 [0.01–0.61] ***p*** **=0.017**	0.01 [0–0.03]	0.01 [0–0.03]	0.01 [0–0.03]
	F0–1	295	11	0.76	0.22 [0.06–0.88] ***p*** **=0.032**	0.02 [0–0.03]	0.03 [0.01–0.05]	0.04 [0.01–0.06]
	Lobular inflammation grade							
	LI2–3	44	6	3.12	Reference	0.09 [0–0.18]	0.12 [0.01–0.21]	0.12 [0.01–0.21]
	LI1	221	7	0.65	0.21 [0.07–0.63] ***p*** **=0.005**	0.02 [0–0.04]	0.04 [0.01–0.06]	0.04 [0.01–0.06]
	LI0	86	3	0.67	0.22 [0.05–0.87] ***p*** **=0.031**	0.01 [0–0.03]	0.03 [0–0.06]	0.05 [0–0.11]
Cirrhosis	Fibrosis stage							
	F3	75	11	3.52	Reference	0.11 [0.04–0.18]	0.15 [0.07–0.23]	0.15 [0.07–0.23]
	F2	95	0	0	NC	0 [0–0]	0 [0–0]	0 [0–0]
	F0–1	295	8	0.54	0.17 [0.07–0.42] ***p*** **<0.001**	0 [0–0]	0.02 [0–0.03]	0.02 [0–0.04]
	Lobular inflammation grade							
	LI2–3	43	5	2.60	Reference	0.05 [0–0.11]	0.1 [0–0.2]	0.1 [0–0.2]
	LI1	212	7	0.66	0.25 [0.08–0.78] ***p*** **=0.018**	0.01 [0–0.03]	0.03 [0.01–0.06]	0.03 [0.01–0.06]
	LI0	84	5	1.20	0.44 [0.13–1.52] *p* **=**0.19	0.02 [0–0.06]	0.05 [0–0.1]	0.07 [0.01–0.12]
Cardiovascular events	Fibrosis stage							
	F4	14	1	1.97	Reference	0.07 [0–0.2]	0.07 [0–0.2]	0.07 [0–0.2]
	F3	70	7	2.11	1.14 [0.13–9.68] *p* **=**0.904	0.03 [0–0.07]	0.03 [0–0.07]	0.09 [0.01–0.17]
	F2	88	4	0.95	0.66 [0.07–6.12] *p* **=**0.712	0 [0–0]	0.01 [0–0.04]	0.06 [0–0.12]
	F0–1	287	10	0.70	0.37 [0.05–3.02] *p* **=**0.355	0 [0–0.01]	0.01 [0–0.02]	0.01 [0–0.02]
	Lobular inflammation grade							
	LI2–3	42	2	1.05	Reference	0 [0–0]	0 [0–0]	0 [0–0]
	LI1	211	9	0.89	0.64 [0.12–3.32] *p* **=**0.595	0 [0–0]	0.01 [0–0.02]	0.03 [0–0.06]
	LI0	81	7	1.75	1.62 [0.30–8.78] *p* **=**0.577	0.03 [0–0.06]	0.04 [0–0.08]	0.06 [0–0.11]
Any long-term outcome	Fibrosis stage							
	F4	14	3	6.86	Reference	0.14 [0–0.31]	0.24 [0–0.44]	0.24 [0–0.44]
	F3	70	13	4.11	0.77 [0.21–2.79] *p* **=**0.687	0.09 [0.02–0.15]	0.09 [0.02–0.15]	0.19 [0.06–0.29]
	F2	88	5	1.21	0.26 [0.06–1.12] *p* **=**0.071	0.01 [0–0.03]	0.03 [0–0.06]	0.07 [0–0.13]
	F0–1	287	18	1.31	0.25 [0.07–0.89] ***p*** **=0.032**	0.02 [0–0.03]	0.03 [0.01–0.05]	0.04 [0.01–0.06]
	Lobular inflammation grade							
	LI2–3	42	7	4.04	Reference	0.07 [0–0.15]	0.1 [0–0.19]	0.1 [0–0.19]
	LI1	211	13	1.31	0.28 [0.11–0.73] ***p*** **=0.009**	0.02 [0–0.04]	0.03 [0.01–0.06]	0.06 [0.02–0.09]
	LI0	81	10	2.60	0.63 [0.24–1.70] *p* **=**0.365	0.04 [0–0.08]	0.07 [0.01–0.12]	0.11 [0.02–0.19]

Significant *p* values are in bold. HRs were adjusted for sex, baseline age, and diabetes status. Association with hepatocyte ballooning was analyzed, but results were inconclusive. Cumulative probabilities for 1-, 3-, and 5-year risk were extracted from Aalen–Johansen curve estimates at the respective timepoints.

Liver-related outcomes were defined as any of the following: HCC, MELD ≥15, hepatic decompensation, liver transplant, or liver-related mortality. Cirrhosis was analyzed separately in patients with F0–3 at baseline and not included in liver-related outcomes. Cardiovascular-related outcomes included any of the following: heart failure, myocardial infarction, ischemic stroke, unstable angina, coronary revascularization, and cardiovascular-related mortality for patients with no cardiovascular outcome events at baseline. Any long-term outcome was defined as any liver-related (excluding cirrhosis) and cardiovascular-related outcomes, and mortality due to liver, cardiovascular, or cancer events.

Abbreviations: F, fibrosis stage; HB, hepatocyte ballooning grade; LI, lobular inflammation grade; MELD, Model for End-Stage Liver Disease score; NC, not calculated; PY, person-years.

### Analysis of long-term outcomes by MASH severity

#### Hepatocyte ballooning and lobular inflammation

Lower grade of hepatocyte ballooning was associated with a lower risk of incident cirrhosis versus higher grade; this was significant for HB1 versus HB2 (HR [95% CI]: 0.20 [0.08–0.50], *p*<0.001) but not HB0 versus HB2 (Figure 4). Incidence rates for HB0 and HB1 were low compared with HB2. No significant associations were identified between hepatocyte ballooning and the risk of liver-related outcomes, and findings regarding the relationship with cardiovascular-related outcomes were inconclusive; there were no significant associations between HB and any outcome (Supplemental Table S6, http://links.lww.com/HC9/A870).

LI1 versus LI2–3 was associated with a significantly lower risk of incident cirrhosis (HR [95% CI]: 0.42 [0.19–0.97], *p* = 0.041) (Figure 4). Although the association between LI and cardiovascular-related outcomes was generally inconclusive, a significant association was identified between reduced risk of stroke and lower lobular inflammation (LI1 vs. LI2–3: HR [95% CI]: 0.13 [0.03–0.50], *p* = 0.003). There was also a significantly lower risk of any outcome with LI1 versus LI2–3 (HR [95% CI]: 0.44 [0.22–0.89], *p* = 0.022) (Supplemental Table S7, http://links.lww.com/HC9/A870).

Analyses of LI stratified by weight-loss surgery revealed that lower (vs. higher) lobular inflammation was associated with lower HRs for liver-related outcomes, including incident cirrhosis, in those patients who had undergone weight-loss surgery (Table 3). Association with hepatocyte ballooning was analyzed but results were inconclusive, albeit with a significantly reduced risk of cirrhosis observed with HB1 versus HB2 in patients without weight-loss surgery (data not shown).

#### Steatosis

No significant associations were identified between steatosis score and incident cirrhosis, liver-related outcomes, cardiovascular-related outcomes, or any outcome. Analyses of the impact of steatosis score on the risk of long-term clinical outcomes stratified by weight-loss surgery were inconclusive.

#### Sensitivity analysis of data after multiple imputations

HBs and LIs were not available for all patients (missing for 26% and 27% of patients, respectively), and 44% of patients did not have complete NAS, so a sensitivity analysis with multiple imputations of missing data was performed. Overall results for NAS with imputed data showed no significant associations between NAS score (low [0–2] and medium [3–4] vs. high [5–8]) and cirrhosis, liver-related outcomes, composite cardiovascular-related outcomes, or any long-term outcome.

Sensitivity analyses of outcomes by LIs and HBs after imputation of missing data gave similar outcomes to the main results, with some additional significant associations (Supplemental Tables S6 and S7, http://links.lww.com/HC9/A870. Sensitivity analysis for HB showed a significant difference in the risk of cirrhosis with HB1 versus HB2 (HR [95% CI]: 0.32 [0.12–0.86]), *p* = 0.025) but not with HB0 versus HB2. There was no significant difference in liver-related outcomes, cardiovascular-related outcomes, or any outcome with HB0 or HB1 versus HB2.

Sensitivity analysis of the association of LI with cirrhosis showed a borderline significant difference in risk of cirrhosis for LI1 versus LI2–3 (HR [95% CI]: 0.44 [0.20–1.00], *p* = 0.050). No significant differences were observed for the composite outcomes—liver-related outcomes, cardiovascular-related outcomes, or any long-term outcomes—with LI0 or LI1 versus LI2–3. However, a significantly reduced risk was identified with LI1 versus LI2–3 for liver decompensation, stroke, and coronary revascularization (also significant with LI0 versus LI2–3) (Supplemental Table S7, http://links.lww.com/HC9/A870).

Sensitivity analysis for steatosis score did not show any significant results for cirrhosis, liver-related outcomes, cardiovascular-related outcomes, any outcome, or individual outcome events (data not shown).

### Exploratory analysis of FIB-4 for liver fibrosis

The number of patients included in this analysis (n = 268) was markedly reduced due to the requirements of repeated FIB-4 measurement availability and exclusion of patients with events for respective outcome analyses before the second measurement date of FIB-4 +30 days. Although there was no statistically significant association between the risk of long-term outcomes and changes in FIB-4 overall, the 360-day change in FIB-4 (HR [95% CI]: 2.04 [0.99–4.19], *p* = 0.053) suggests a potential association that could be analyzed robustly in a cohort with an increased sample size. Multivariable modeling suggested that the risk of liver-related outcomes was influenced by the baseline FIB-4 value (higher risk with higher value at baseline, Supplemental Table S8, http://links.lww.com/HC9/A870).

## DISCUSSION

This study provides real-world support for previously published data showing that the severity of fibrosis is associated with the risk of liver-related outcomes including mortality.^8–11,20–23^ Although fibrosis stage was the only histological parameter found to be significantly associated with the composite endpoint of risk of any liver-related outcome (HCC, Model for End-Stage Liver Disease score ≥15, liver transplant, and hepatic decompensation events), lower levels of hepatocyte ballooning and liver inflammation were associated with a lower risk of incident cirrhosis. This suggests that hepatocyte ballooning and liver inflammation drive disease activity and ultimately can promote hepatic fibrosis progression. If so, reversing these changes earlier in the disease course could potentially reduce the likelihood of progression to cirrhosis or other serious long-term outcomes. However, this needs to be prospectively investigated as it cannot easily be demonstrated using real-world database studies in which repeat biopsy data are often lacking. Nevertheless, in the absence of such data, these current findings highlight the potential of hepatocyte ballooning and liver inflammation (assessed at baseline biopsy) as surrogate markers of disease progression.

Lower versus higher fibrosis stages were associated with a lower rate of both liver events and cardiovascular-related outcomes. This is consistent with previous studies where MASH and hepatic fibrosis were associated with an increased risk of liver-related mortality as well as atherosclerotic cardiovascular disease after adjustment for classical cardiovascular disease risk factors.^2,8,9,20–22,24^ Generally, associations between liver inflammation and cardiovascular-related outcomes were inconclusive in our study. However, lower liver inflammation was associated with a reduced risk of stroke. One can postulate that systemic inflammation may mediate fibrosis in both MASLD and coronary artery disease.^24^ However, given the low rate of cardiovascular-related outcomes in the current analysis and the relatively small sample size, further research is warranted.

Approximately two-thirds of individuals in the current study had undergone weight-loss surgery on or before the index date. This compares with ~10% of individuals with MASLD in the SD database overall. Furthermore, individuals in our study were selected for having undergone a liver biopsy and, in the SD database, people with liver biopsies were much more likely than those without biopsies to have a history of weight-loss surgery. This suggests that biopsies performed in real-life settings in the United States may, to a large extent, emanate from patients undergoing weight-loss surgery; this should be actively investigated as a parameter in future studies. In addition, it suggests that our results may be more generalizable to patients eligible for weight-loss surgery. Weight-loss surgery has been proven to lower the risk of cardiovascular events, cardiovascular death, and overall mortality.^25–27^ In our study, incidence rates for long-term liver-related and cardiovascular-related outcomes appeared to be lower overall in patients who underwent weight-loss surgery when compared with those without weight-loss surgery. However, without formal comparisons, it is not possible to conclude whether these apparent differences are statistically significant. It is also not possible to conclude from this study whether the apparent lower event rate in those who underwent weight-loss surgery was attributable to the putative beneficial effects of weight-loss surgery. Nevertheless, patterns of associations between lower (vs. higher) fibrosis stages and lower risks for liver-related outcomes including cirrhosis remained regardless of weight-loss surgery status, as shown by adjustment in the main analysis and additionally in subgroup analyses. Because the sample size of patients without weight-loss surgery was limited and the number of events was low, the presence of a potential bias cannot be excluded; therefore, weight loss may still have an effect on long-term outcomes. Nevertheless, it is worth noting that in a previous study, patients with severe MASLD had a greater than 3-fold risk of death following weight-loss surgery, suggesting that the risk of outcomes may be associated with MASLD, even after weight-loss surgery.^28^

There were no clear associations between long-term outcomes and change in FIB-4 at 360 days after biopsy, but overall patterns were supportive of the primary results. These results may be considered directional, which might be expected given the relatively low number of patients and events available for the analysis.

The real-world nature of this study reflects the risk of outcomes in clinical practice in patients with and without weight-loss surgery. The real-world design offered the advantage of allowing investigation of the impact of MASH across a breadth of liver-related and cardiovascular-related outcomes. Furthermore, the overall sample size of 702 patients is larger than most previous individual studies that assessed multiple liver-related and cardiovascular-related outcomes in MASLD including MASH, although 1 meta-analysis involving multiple trials provided additional statistical power to detect outcomes.^9,20,22^ In the current study, we identified a “MASH-like” population according to pathologist judgement, reflecting real-world histological assessments. Although other studies have looked at outcomes in a broader set of patients with MASLD, this approach has the drawback of including patients with much less severe disease. In contrast, patients without “likely MASH” were excluded from the current analyses. This potentially gives a more accurate estimate of outcome risks with a focus on patients identified as MASH in real-world practice, and perhaps better reflects the patient population eligible for clinical trials. While previous studies have assessed the association between fibrosis and long-term outcomes, these comparisons were done in patients with fibrosis versus patients with no or mild–moderate fibrosis^8–11,20–23^; in contrast, the present study compared long-term outcomes in patients with no or mild–moderate fibrosis versus those with advanced disease (cirrhosis) to assess the benefit of halting disease progression. The fact that outcomes were analyzed with and without imputation of missing data can also be considered a strength, as can the high proportion of patients with weight-loss surgery in the database. The latter reflects a real-world cohort, but it may not be representative of other populations outside the region studied, which may have different levels of weight-loss surgery.

The study has some limitations. The number of outcome events was relatively low, which may have masked associations between histological parameters and outcomes. There were also small sample sizes for some analyses, particularly of individual cardiovascular events, leading to noncalculable adjusted HRs and wide CIs for several outcomes. Sample size limitations also prevented stratification of hepatocyte ballooning and liver inflammation by fibrosis stage, which could have provided more granularity on the extent to which greater severity in these features was potentially mediated by increased fibrosis. The considerable extent of interobserver and intraobserver variability in pathologist interpretations of liver biopsies, particularly regarding hepatocyte ballooning and liver inflammation,^29^ is acknowledged as a limitation. Analyses of liver biopsy data were impacted by the lack of access to original histology slides, with only the reports available. Most liver biopsy reports do not provide a clear and direct estimate of NAS or grade/score of hepatocyte ballooning, liver inflammation, and steatosis; therefore, assumptions were made to standardize the application of a retrospective scoring system based solely on the information in the pathology report (review criteria in the Supplemental Digital Content, http://links.lww.com/HC9/A870). For example, in some patients, a score or grade of zero for hepatocyte ballooning or liver inflammation may have been interpreted as low or borderline ballooning or inflammation and thus mentioned in the biopsy report as “steatohepatitis.” The timing of biopsies was also not standardized and can occur at various points during the clinical care of each patient with MASLD. Patients with diagnostic evidence of liver conditions other than MASLD during both baseline and follow-up were excluded from the analysis. This was implemented to reduce the confounding effects of other liver conditions on long-term outcomes. Diagnostic evidence of other liver conditions was based on ICD codes, but reporting of diagnoses can vary across clinicians and across the spectrum of clinical concern for MASLD. For example, abnormalities in the patient’s liver enzyme labs may indicate a liver biopsy with evidence of steatosis, but ICD codes for alcohol abuse are not evident in the patient’s EMR until several days after the biopsy. While this approach addresses these ambiguities in coding and increases the confidence that the effects on long-term outcomes are related to MASH, we acknowledge that it differs from real-world clinical practice, where it is not possible to predict whether a patient will develop future liver disease at the time of first biopsy, and liver-related outcomes may further be affected due to other liver conditions. In addition, the database used in this study is exclusive to patients seeking care with Vanderbilt University Medical Center, and thus reflects the predominant demographics of patients in the southeastern United States and the standards of care at that medical center. In the US health care system, patients may not receive all their care at a single facility, and data outside of the medical center’s system are limited or not available. This limitation is particularly relevant for mortality data because deaths may have occurred elsewhere than the medical center. Further studies using data from different geographical regions with different health care systems and clinical practices are warranted to generalize the findings to the global population.

Finally, although not a limitation per se, it should be noted that we did not include a comparison cohort of patients without MASH because our focus was on long-term outcomes in a specifically MASH-like population. Importantly, although our analysis uses the new MASH/MASLD nomenclature,^30^ diagnoses were based on clinical outcomes defined by ICD codes for NASH/NAFLD.

Overall, our findings from real-world practice support existing published tertiary care data on the importance of liver histology in signaling long-term risk of adverse outcomes in people with MASH. We confirm that stage of fibrosis is predictive of adverse outcomes, and show that lower liver inflammation and HBs are associated with a lower risk of incident cirrhosis, highlighting the relevance of these histologic features in predicting long-term outcomes.

## Supplementary Material
