# Supplementary material for: Liver histology is associated with long-term clinical outcomes in patients with metabolic dysfunction–associated steatohepatitis
Source: Hepatol Commun. 2024 May 10;8(6):e0423. doi: 10.1097/HC9.0000000000000423 (PMC11093565; doi:10.1097/HC9.0000000000000423)
Supplement: Supplementary file 2 [file hc9-8-e0423-s002.pdf]

ICD Code	ICD Description	Disease Correlate	ICD Code Type
291.0	Alcohol withdrawal delirium	Alcohol	ICD9
291.1	Alcohol-induced persisting amnestic disorder	Alcohol	ICD9
291.2	Alcohol-induced persisting dementia	Alcohol	ICD9
291.3	Alcohol-induced psychotic disorder with hallucinations	Alcohol	ICD9
291.4	Idiosyncratic alcohol intoxication	Alcohol	ICD9
291.5	Alcohol-induced psychotic disorder with delusions	Alcohol	ICD9
291.81	Alcohol withdrawal	Alcohol	ICD9
291.82	Alcohol induced sleep disorders	Alcohol	ICD9
291.89	Other alcohol-induced mental disorders	Alcohol	ICD9
291.9	Unspecified alcohol-induced mental disorders	Alcohol	ICD9
303.00	Acute alcoholic intoxication in alcoholism, unspecified	Alcohol	ICD9
303.01	Acute alcoholic intoxication in alcoholism, continuous	Alcohol	ICD9
303.02	Acute alcoholic intoxication in alcoholism, episodic	Alcohol	ICD9
303.03	Acute alcoholic intoxication in alcoholism, in remission	Alcohol	ICD9
303.90	Other and unspecified alcohol dependence, unspecified	Alcohol	ICD9
303.91	Other and unspecified alcohol dependence, continuous	Alcohol	ICD9
303.92	Other and unspecified alcohol dependence, episodic	Alcohol	ICD9
303.93	Other and unspecified alcohol dependence, in remission	Alcohol	ICD9
305.00	Alcohol abuse, unspecified	Alcohol	ICD9
305.01	Alcohol abuse, continuous	Alcohol	ICD9
305.02	Alcohol abuse, episodic	Alcohol	ICD9
305.03	Alcohol abuse, in remission	Alcohol	ICD9
357.5	Alcoholic polyneuropathy	Alcohol	ICD9
425.5	Alcoholic cardiomyopathy	Alcohol	ICD9
535.30	Alcoholic gastritis, without mention of hemorrhage	Alcohol	ICD9
535.31	Alcoholic gastritis, with hemorrhage	Alcohol	ICD9
571.0	Alcoholic fatty liver	Alcohol	ICD9
571.1	Acute alcoholic hepatitis	Alcohol	ICD9
571.2	Alcoholic cirrhosis of liver	Alcohol	ICD9
571.3	Alcoholic liver damage, unspecified	Alcohol	ICD9
V11.3	Personal history of alcoholism	Alcohol	ICD9
E24.4	Alcohol-induced pseudo-Cushing's syndrome	Alcohol	ICD10
F10.10	Alcohol abuse, uncomplicated	Alcohol	ICD10
F10.11	Alcohol abuse, in remission	Alcohol	ICD10
F10.120	Alcohol abuse with intoxication, uncomplicated	Alcohol	ICD10
F10.121	Alcohol abuse with intoxication delirium	Alcohol	ICD10
F10.129	Alcohol abuse with intoxication, unspecified	Alcohol	ICD10
F10.130	Alcohol abuse with withdrawal, uncomplicated	Alcohol	ICD10
F10.131	Alcohol abuse with withdrawal delirium	Alcohol	ICD10
F10.132	Alcohol abuse with withdrawal with perceptual disturbance	Alcohol	ICD10
F10.139	Alcohol abuse with withdrawal, unspecified	Alcohol	ICD10
F10.14	Alcohol abuse with alcohol-induced mood disorder	Alcohol	ICD10
F10.150	Alcohol abuse with alcohol-induced psychotic disorder with delusions	Alcohol	ICD10
F10.151	Alcohol abuse with alcohol-induced psychotic disorder with hallucinations	Alcohol	ICD10
F10.159	Alcohol abuse with alcohol-induced psychotic disorder, unspecified	Alcohol	ICD10
F10.180	Alcohol abuse with alcohol-induced anxiety disorder	Alcohol	ICD10
F10.181	Alcohol abuse with alcohol-induced sexual dysfunction	Alcohol	ICD10
F10.182	Alcohol abuse with alcohol-induced sleep disorder	Alcohol	ICD10
F10.188	Alcohol abuse with other alcohol-induced disorder	Alcohol	ICD10
F10.19	Alcohol abuse with unspecified alcohol-induced disorder	Alcohol	ICD10
F10.20	Alcohol dependence, uncomplicated	Alcohol	ICD10
F10.21	Alcohol dependence, in remission	Alcohol	ICD10
F10.220	Alcohol dependence with intoxication, uncomplicated	Alcohol	ICD10
F10.221	Alcohol dependence with intoxication delirium	Alcohol	ICD10
F10.229	Alcohol dependence with intoxication, unspecified	Alcohol	ICD10
F10.230	Alcohol dependence with withdrawal, uncomplicated	Alcohol	ICD10
F10.231	Alcohol dependence with withdrawal delirium	Alcohol	ICD10
F10.232	Alcohol dependence with withdrawal with perceptual disturbance	Alcohol	ICD10
F10.239	Alcohol dependence with withdrawal, unspecified	Alcohol	ICD10
F10.24	Alcohol dependence with alcohol-induced mood disorder	Alcohol	ICD10
F10.250	Alcohol dependence with alcohol-induced psychotic disorder with delusions	Alcohol	ICD10
F10.251	Alcohol dependence with alcohol-induced psychotic disorder with hallucinations	Alcohol	ICD10
F10.259	Alcohol dependence with alcohol-induced psychotic disorder, unspecified	Alcohol	ICD10
F10.26	Alcohol dependence with alcohol-induced persisting amnestic disorder	Alcohol	ICD10
F10.27	Alcohol dependence with alcohol-induced persisting dementia	Alcohol	ICD10
F10.280	Alcohol dependence with alcohol-induced anxiety disorder	Alcohol	ICD10
F10.281	Alcohol dependence with alcohol-induced sexual dysfunction	Alcohol	ICD10
F10.282	Alcohol dependence with alcohol-induced sleep disorder	Alcohol	ICD10
F10.288	Alcohol dependence with other alcohol-induced disorder	Alcohol	ICD10
F10.29	Alcohol dependence with unspecified alcohol-induced disorder	Alcohol	ICD10
F10.920	Alcohol use, unspecified with intoxication, uncomplicated	Alcohol	ICD10
F10.921	Alcohol use, unspecified with intoxication delirium	Alcohol	ICD10
F10.929	Alcohol use, unspecified with intoxication, unspecified	Alcohol	ICD10
F10.930	Alcohol use, unspecified with withdrawal, uncomplicated	Alcohol	ICD10
F10.931	Alcohol use, unspecified with withdrawal delirium	Alcohol	ICD10
F10.932	Alcohol use, unspecified with withdrawal with perceptual disturbance	Alcohol	ICD10
F10.939	Alcohol use, unspecified with withdrawal, unspecified	Alcohol	ICD10
F10.94	Alcohol use, unspecified with alcohol-induced mood disorder	Alcohol	ICD10
F10.950	Alcohol use, unspecified with alcohol-induced psychotic disorder with delusions	Alcohol	ICD10
F10.951	Alcohol use, unspecified with alcohol-induced psychotic disorder with hallucinations	Alcohol	ICD10
F10.959	Alcohol use, unspecified with alcohol-induced psychotic disorder, unspecified	Alcohol	ICD10
F10.96	Alcohol use, unspecified with alcohol-induced persisting amnestic disorder	Alcohol	ICD10
F10.97	Alcohol use, unspecified with alcohol-induced persisting dementia	Alcohol	ICD10
F10.980	Alcohol use, unspecified with alcohol-induced anxiety disorder	Alcohol	ICD10
F10.981	Alcohol use, unspecified with alcohol-induced sexual dysfunction	Alcohol	ICD10
F10.982	Alcohol use, unspecified with alcohol-induced sleep disorder	Alcohol	ICD10
F10.988	Alcohol use, unspecified with other alcohol-induced disorder	Alcohol	ICD10
F10.99	Alcohol use, unspecified with unspecified alcohol-induced disorder	Alcohol	ICD10
G31.2	Degeneration of nervous system due to alcohol	Alcohol	ICD10
G62.1	Alcoholic polyneuropathy	Alcohol	ICD10
G72.1	Alcoholic myopathy	Alcohol	ICD10
I42.6	Alcoholic cardiomyopathy	Alcohol	ICD10
K29.20	Alcoholic gastritis without bleeding	Alcohol	ICD10
K29.21	Alcoholic gastritis with bleeding	Alcohol	ICD10
K70.0	Alcoholic fatty liver	Alcohol	ICD10
K70.10	Alcoholic hepatitis without ascites	Alcohol	ICD10
K70.11	Alcoholic hepatitis with ascites	Alcohol	ICD10
K70.2	Alcoholic fibrosis and sclerosis of liver	Alcohol	ICD10
K70.30	Alcoholic cirrhosis of liver without ascites	Alcohol	ICD10
K70.31	Alcoholic cirrhosis of liver with ascites	Alcohol	ICD10
K70.40	Alcoholic hepatic failure without coma	Alcohol	ICD10
K70.41	Alcoholic hepatic failure with coma	Alcohol	ICD10
K70.9	Alcoholic liver disease, unspecified	Alcohol	ICD10
K85.20	Alcohol induced acute pancreatitis without necrosis or infection	Alcohol	ICD10
K85.21	Alcohol induced acute pancreatitis with uninfected necrosis	Alcohol	ICD10
K85.22	Alcohol induced acute pancreatitis with infected necrosis	Alcohol	ICD10
K86.0	Alcohol-induced chronic pancreatitis	Alcohol	ICD10
O35.4XX0	Maternal care for (suspected) damage to fetus from alcohol, not applicable or unspecified	Alcohol	ICD10
O35.4XX1	Maternal care for (suspected) damage to fetus from alcohol, fetus 1	Alcohol	ICD10
O35.4XX2	Maternal care for (suspected) damage to fetus from alcohol, fetus 2	Alcohol	ICD10
O35.4XX3	Maternal care for (suspected) damage to fetus from alcohol, fetus 3	Alcohol	ICD10
O35.4XX4	Maternal care for (suspected) damage to fetus from alcohol, fetus 4	Alcohol	ICD10
O35.4XX5	Maternal care for (suspected) damage to fetus from alcohol, fetus 5	Alcohol	ICD10
O35.4XX9	Maternal care for (suspected) damage to fetus from alcohol, other fetus	Alcohol	ICD10
O99.310	Alcohol use complicating pregnancy, unspecified trimester	Alcohol	ICD10
O99.311	Alcohol use complicating pregnancy, first trimester	Alcohol	ICD10
O99.312	Alcohol use complicating pregnancy, second trimester	Alcohol	ICD10
O99.313	Alcohol use complicating pregnancy, third trimester	Alcohol	ICD10
O99.314	Alcohol use complicating childbirth	Alcohol	ICD10
O99.315	Alcohol use complicating the puerperium	Alcohol	ICD10
Z71.41	Alcohol abuse counseling and surveillance of alcoholic	Alcohol	ICD10
571.42	Autoimmune hepatitis	Autoimmune	ICD9
K75.4	Autoimmune hepatitis	Autoimmune	ICD10
571.6	Biliary cirrhosis	Biliary cirrhosis	ICD9
K74.3	Primary biliary cirrhosis	Biliary cirrhosis	ICD10
K74.4	Secondary biliary cirrhosis	Biliary cirrhosis	ICD10
K74.5	Biliary cirrhosis, unspecified	Biliary cirrhosis	ICD10
576.1	Cholangitis	Cholangitis	ICD9
K83.01	Primary sclerosing cholangitis	Cholangitis	ICD10
K83.09	Other cholangitis	Cholangitis	ICD10
275.01	Hereditary hemochromatosis	Hemochromatosis	ICD9
275.02	Hemochromatosis due to repeated red blood cell transfusions	Hemochromatosis	ICD9
275.03	Other hemochromatosis	Hemochromatosis	ICD9
E83.110	Hereditary hemochromatosis	Hemochromatosis	ICD10
E83.111	Hemochromatosis due to repeated red blood cell transfusions	Hemochromatosis	ICD10
E83.118	Other hemochromatosis	Hemochromatosis	ICD10
E83.119	Hemochromatosis, unspecified	Hemochromatosis	ICD10
573.1	Hepatitis in viral diseases classified elsewhere	Other liver	ICD9
573.2	Hepatitis in other infectious diseases classified elsewhere	Other liver	ICD9
K77	Liver disorders in diseases classified elsewhere	Other liver	ICD10
K71.0	Toxic liver disease with cholestasis	Toxic liver	ICD10
K71.10	Toxic liver disease with hepatic necrosis, without coma	Toxic liver	ICD10
K71.11	Toxic liver disease with hepatic necrosis, with coma	Toxic liver	ICD10
K71.2	Toxic liver disease with acute hepatitis	Toxic liver	ICD10
K71.3	Toxic liver disease with chronic persistent hepatitis	Toxic liver	ICD10
K71.4	Toxic liver disease with chronic lobular hepatitis	Toxic liver	ICD10
K71.50	Toxic liver disease with chronic active hepatitis without ascites	Toxic liver	ICD10
K71.51	Toxic liver disease with chronic active hepatitis with ascites	Toxic liver	ICD10
K71.6	Toxic liver disease with hepatitis, not elsewhere classified	Toxic liver	ICD10
K71.7	Toxic liver disease with fibrosis and cirrhosis of liver	Toxic liver	ICD10
K71.8	Toxic liver disease with other disorders of liver	Toxic liver	ICD10
K71.9	Toxic liver disease, unspecified	Toxic liver	ICD10
070.0	Viral hepatitis A with hepatic coma	Viral hepatitis	ICD9
070.1	Viral hepatitis A without mention of hepatic coma	Viral hepatitis	ICD9
070.20	Viral hepatitis B with hepatic coma, acute or unspecified, without mention of hepatitis delta	Viral hepatitis	ICD9
070.21	Viral hepatitis B with hepatic coma, acute or unspecified, with hepatitis delta	Viral hepatitis	ICD9
070.22	Chronic viral hepatitis B with hepatic coma without hepatitis delta	Viral hepatitis	ICD9
070.23	Chronic viral hepatitis B with hepatic coma with hepatitis delta	Viral hepatitis	ICD9
070.30	Viral hepatitis B without mention of hepatic coma, acute or unspecified, without mention of hepatitis delta	Viral hepatitis	ICD9
070.31	Viral hepatitis B without mention of hepatic coma, acute or unspecified, with hepatitis delta	Viral hepatitis	ICD9
070.32	Chronic viral hepatitis B without mention of hepatic coma without mention of hepatitis delta	Viral hepatitis	ICD9
070.33	Chronic viral hepatitis B without mention of hepatic coma with hepatitis delta	Viral hepatitis	ICD9
070.41	Acute hepatitis C with hepatic coma	Viral hepatitis	ICD9
070.42	Hepatitis delta without mention of active hepatitis B disease with hepatic coma	Viral hepatitis	ICD9
070.43	Hepatitis E with hepatic coma	Viral hepatitis	ICD9
070.44	Chronic hepatitis C with hepatic coma	Viral hepatitis	ICD9
070.49	Other specified viral hepatitis with hepatic coma	Viral hepatitis	ICD9
070.51	Acute hepatitis C without mention of hepatic coma	Viral hepatitis	ICD9
070.52	Hepatitis delta without mention of active hepatitis B disease or hepatic coma	Viral hepatitis	ICD9
070.53	Hepatitis E without mention of hepatic coma	Viral hepatitis	ICD9
070.54	Chronic hepatitis C without mention of hepatic coma	Viral hepatitis	ICD9
070.59	Other specified viral hepatitis without mention of hepatic coma	Viral hepatitis	ICD9
070.6	Unspecified viral hepatitis with hepatic coma	Viral hepatitis	ICD9
070.70	Unspecified viral hepatitis C without hepatic coma	Viral hepatitis	ICD9
070.71	Unspecified viral hepatitis C with hepatic coma	Viral hepatitis	ICD9
070.9	Unspecified viral hepatitis without mention of hepatic coma	Viral hepatitis	ICD9
B15.0	Hepatitis A with hepatic coma	Viral hepatitis	ICD10
B15.9	Hepatitis A without hepatic coma	Viral hepatitis	ICD10
B16.0	Acute hepatitis B with delta-agent with hepatic coma	Viral hepatitis	ICD10
B16.1	Acute hepatitis B with delta-agent without hepatic coma	Viral hepatitis	ICD10
B16.2	Acute hepatitis B without delta-agent with hepatic coma	Viral hepatitis	ICD10
B16.9	Acute hepatitis B without delta-agent and without hepatic coma	Viral hepatitis	ICD10
B17.0	Acute delta-(super) infection of hepatitis B carrier	Viral hepatitis	ICD10
B17.10	Acute hepatitis C without hepatic coma	Viral hepatitis	ICD10
B17.11	Acute hepatitis C with hepatic coma	Viral hepatitis	ICD10
B17.2	Acute hepatitis E	Viral hepatitis	ICD10
B17.8	Other specified acute viral hepatitis	Viral hepatitis	ICD10
B17.9	Acute viral hepatitis, unspecified	Viral hepatitis	ICD10
B18.0	Chronic viral hepatitis B with delta-agent	Viral hepatitis	ICD10
B18.1	Chronic viral hepatitis B without delta-agent	Viral hepatitis	ICD10
B18.2	Chronic viral hepatitis C	Viral hepatitis	ICD10
B18.8	Other chronic viral hepatitis	Viral hepatitis	ICD10
B18.9	Chronic viral hepatitis, unspecified	Viral hepatitis	ICD10
B19.0	Unspecified viral hepatitis with hepatic coma	Viral hepatitis	ICD10
B19.10	Unspecified viral hepatitis B without hepatic coma	Viral hepatitis	ICD10
B19.11	Unspecified viral hepatitis B with hepatic coma	Viral hepatitis	ICD10
B19.20	Unspecified viral hepatitis C without hepatic coma	Viral hepatitis	ICD10
B19.21	Unspecified viral hepatitis C with hepatic coma	Viral hepatitis	ICD10
B19.9	Unspecified viral hepatitis without hepatic coma	Viral hepatitis	ICD10
275.1	Disorders of copper metabolism	Wilson's disease	ICD9
E83.01	Wilson's disease	Wilson's disease	ICD10
