# Supplementary material for: Liver histology is associated with long-term clinical outcomes in patients with metabolic dysfunction–associated steatohepatitis
Source: Hepatol Commun. 2024 May 10;8(6):e0423. doi: 10.1097/HC9.0000000000000423 (PMC11093565; doi:10.1097/HC9.0000000000000423)
Supplement: Supplementary file 3 [file hc9-8-e0423-s003.pdf]

Liver histology is associated with long-term clinical outcomes in patients with metabolic dysfunction-associated steatohepatitis

Zobair M. Younossi MD,[1,2] Kamal Kant Mangla BTech,[3] Tina Landsvig Berentzen PhD,[3] Katrine Grau PhD,[3] Mette Skalshøi Kjær MD,[3] Steen Ladelund,[3] Louise Maymann Nitze PhD,[3] Crystal Coolbaugh PhD,[4] Chih-Yuan Hsu PhD,[4] Hannes Hagström MD, PhD[5,6]

[1]Beatty Liver and Obesity Research Program, Inova Health System, Falls Church, VA, USA; [2]The Global NASH Council, Washington DC, USA; [3]Novo Nordisk A/S, Søborg, Denmark; [4]Nashville Biosciences, Nashville, TN, USA; [5]Department of Medicine, Huddinge, Karolinska Institutet, Stockholm, Sweden; [6]Division of Hepatology, Karolinska University Hospital, Stockholm, Sweden.

SUPPLEMENTARY APPENDIX

Table of Contents

Histology evaluation criteria 2
Detailed statistical analysis methods 5
List of ICD and CPT codes 8
List of exclusion ICD codes 9
SDC TABLE 1. Additional baseline characteristics (NAS and components) stratified by baseline stages of fibrosis. 10
SDC TABLE 2. Key baseline characteristics stratified by baseline grades of hepatocyte ballooning. 12
SDC TABLE 3. Key baseline characteristics stratified by baseline grades of lobular inflammation. 13
SDC TABLE 4. Key baseline characteristics stratified by baseline scores of steatosis. 14
SDC TABLE 5. Hazard ratios and incidence rates for liver-related outcomes, incident cirrhosis, and cardiovascular-related outcomes, stratified by baseline fibrosis stage (overall and individual events). 15
SDC TABLE 6. Hazard ratios and incidence rates for liver-related outcomes, incident cirrhosis, and cardiovascular-related outcomes, stratified by baseline hepatocyte ballooning grade (overall and individual events). 20
SDC TABLE 7. Hazard ratios and incidence rates for liver-related outcomes, incident cirrhosis, and cardiovascular-related outcomes, stratified by baseline lobular inflammation grade (overall and individual events). 24

SDC TABLE 8. Exploratory analysis of FIB-4: adjusted hazard ratios for liver-related outcomes. 28

Histology evaluation criteria

Excluded pathology reports:

- Pending addendum: preliminary report (scoring performed on final report)
- Non-liver: self-explanatory
- Liver – tumor (without steatosis): biopsy for presence or absence of tumor
- Duplicate: self-explanatory
- Frozen section: intraoperative consultation prior to final report (not formalin-fixed, paraffin-embedded tissue)
- Cytology: lacks architectural detail
- Addendum: report separate from primary report
    - Separate addendum reports (when present and relevant) are copied to the comment section of the primary report
- Transplant (without steatosis): biopsy assessing rejection status
- Cytogenetics: not histology
- Neonatal: biopsy of newborn liver
- Electron microscopy: not used for MASLD scoring
- Mutation analysis: not histology

Biopsy interpretation assumptions:

- Standard scale for lobular inflammation and steatosis is mild/moderate/severe (or marked)
- If range is given (e.g. mild to moderate), then scoring based on more severe end of range
- Negative for/no steatohepatitis = no lobular inflammation or hepatocyte ballooning
- Minimal/focal/rare/minute/small/little understood to mean mild/few across all scales
- Diffuse understood to mean severe (or marked)
- Pericellular/perisinusoidal/perivenular considered roughly equivalent terms

- Septal fibrosis mentioned by itself = portal extension
- Activity = lobular inflammation (but must specify lobular activity or be associated with description of steatohepatitis)
- If hepatitis grade reported, then 1-2 = minimal-mild, 3 = moderate, 4 = severe
- No hepatitis = no inflammation
- Steatosis without active…/without activity… = no lobular inflammation or hepatocyte ballooning
- No hepatocyte injury/hepatocytes without damage = no hepatocyte ballooning
- No significant histopathologic change (or equivalent) = no abnormalities for any finding not otherwise addressed

Biopsy interpretation qualifier key:

i - Parameter not reported and no further clinical determination possible

ii - Parameter not reported but clinical inference possible

iii - Parameter reported but not sufficiently described to directly apply to a scale - clinical judgement required

- Steatosis not mentioned but steatohepatitis is = steatosis of 1 with qualifier of iii

iv - Parameter reported but described in a non-standardized fashion - clinical judgement required

- Steatosis: patchy = mild, scattered = moderate
- lobular inflammation: spotty/patchy = mild, occasional/scattered = moderate
- hepatocyte ballooning: occasional/scattered/some/spotty = few, frequent/moderate/well-developed = many
- Zone 3 fibrosis: spotty/patchy/occasional/scattered = mild

Biopsy categorization (more likely MASH vs. more likely MASL):

1. Any biopsy with at least 5% steatosis will be considered for inclusion
    - Biopsies with no steatosis are excluded *(biopsies with no steatosis but high fibrosis could be considered as possible end-stage MASH)*
    - If steatohepatitis or MASLD/NAFLD is mentioned in the report, then biopsy is included regardless of amount of steatosis

2. Any biopsy with obvious mention of other causes of steatosis or steatohepatitis will be excluded (e.g. methotrexate use, Wilson disease, etc.)
3. Any biopsy mentioning "steatohepatitis" will be placed in the more likely MASH category

- Any biopsy mentioning "borderline steatohepatitis" will be placed in the more likely MASH category
- Any biopsy mentioning "not steatohepatitis" or equivalent will be placed in the more likely MASL category

4. Any biopsy with NAS score of 5 or higher will be placed in the more likely MASH category
5. Any biopsy (with NAS score of less than 5) demonstrating both hepatocyte ballooning degeneration and pericellular/perisinusoidal fibrosis will be placed in the more likely MASH category *(biopsies with only pericellular/perisinusoidal fibrosis or only hepatocyte ballooning degeneration could be considered as more likely MASH)*
6. Any biopsy with evidence of severe fibrosis/cirrhosis will be placed in the more likely MASH category
7. Any biopsy lacking features 3-6 will be placed in the more likely MASL category

Detailed statistical analysis methods

General considerations

If not otherwise specified, the main analysis and Fibrosis-4 index (FIB-4) analysis were done on all patients in the analysis set, whereas analyses for cirrhosis events were done on the subpopulation with fibrosis stage F0 to F3 at baseline and no cirrhosis events prior to the index date. All statistical analyses were performed using R version 4.0.3, and the R packages survival 3.2-7, survminer 0.4.9, xtable 1.8-4, and Hmisc 4.4.2.

Significance level

A significance level of 0.05 for 2-sided comparisons was considered statistically significant, for example, $p<0.05$ in log-rank test. HRs and 95% CIs were derived. The differences in time to events were also treated as statistically significant if 95% CIs did not cross one.

Presentation of continuous and qualitative variables

Descriptive statistics derived for continuous variables (e.g., age and BMI) were reported as median (IQR). Qualitative variables were summarized by counts and percentages based on the number of patients in the analysis of interest, unless otherwise specified.

Definition of index event

The date of the first liver biopsy pathology report with histologic evidence of MASH was used as the index event for each patient. Fibrosis stage, total NAS score, and the NAS score subcomponents (lobular inflammation, hepatocyte ballooning, steatosis) at baseline were defined using the information documented in the pathology report and according to the review criteria outlined in the histology evaluation criteria section above.

Definition of event

If not otherwise specified, patient records were surveyed from the date of the index event until the most recent EMR data entry to identify the first incidence of a clinical event of interest. This was based on the assumption that the patient did not have a pre-existing diagnosis of the clinical outcome prior to receiving health care within the Vanderbilt University Medical Center system. The validity of this assumption was checked for each event by evaluating the number of events in close proximity to the index date and excluding patients with pre-existing conditions accordingly (e.g., with the first event incidence occurring within 30 days of the index date). The time to event (i.e., time to first diagnosis) was calculated as event date minus index date.

For the analysis focusing on change in FIB-4 from baseline to 360 days (±90 days) follow-up, time to event was calculated as event date minus the date 30 days past the date of the 360-day FIB-4 follow-up.

Main analyses

Patients with pre-existing cardiovascular events prior to the liver biopsy index date were excluded from analysis of both cardiovascular-related events and overall diagnostic incidence. In the main analysis, death from any cause was considered as a competing event of any non-fatal event. The censoring observations caused by deaths happening before the non-fatal events were not assumed to be independent of the event time. The Aalen-Johansen method with death as a competing risk was used to estimate the cumulative probabilities of non-fatal events for each subtype of fibrosis stages and baseline liver-related histological parameters (the cumulative probability curves with zero events were not plotted), and the Fine-Gray competing risk model was used to estimate the sub-distribution hazard ratios between subtypes. For the total outcome event, the Cox proportional hazards model was used, where the mortality status at end of follow-up was treated as censored rather than missing if no mortality occurred during follow-up. The score (log rank) test was used to compare the hazards between subtypes, and the p-value presented to show significance (if $p < 0.05$). Also, the HRs without and with adjustment for covariates were shown, denoted as the crude HRs and the adjusted HRs, respectively, along with the 95% CIs. The covariates used in Fine-Gray competing risk or Cox models included baseline age, gender, baseline diabetes, and baseline weight loss surgery. In the analyses for cirrhosis, HCC, MELD score, and decompensation events, one case with liver transplant was excluded.

Using similar statistical methodology to the main analyses, HRs and 95% CIs were calculated with covariates as change in FIB-4 from baseline, age at baseline, gender, baseline diabetes status, weight loss surgery status at baseline and respective FIB-4 value at baseline (baseline refers to the index date defined as first identified biopsy). Results from the multivariable modelling were also used to understand the effect on outcomes by other covariates.

Missing data and imputation rules

In the analysis of association of time to events with NAS score, which is the summation of three baseline liver-related histological parameters (steatosis, lobular inflammation, and hepatocyte ballooning), multiple imputation (with five imputations) for missing values of baseline liver-related histological parameters was performed through additive regression, bootstrapping, and

predictive mean matching. The imputation did not include missing time-to-outcome and censored status. The final results were the average of the five imputed datasets. The multiple imputation approach took all aspects of uncertainty of the imputations into account by using bootstrap to approximate the process of drawing predicted values from a full Bayesian (posterior) predictive distribution. After imputations, the Fine-Gray competing risk or Cox model was applied to each of the five imputed datasets. The analysis results were different for each imputed dataset due to the randomness of the predicted values, and there was a variance between the results. Incorporating the results with the variance, the average results were reported, along with the 95% CIs.

FIB-4 analyses

The baseline measurement (including FIB-4 index) was defined as the measurement occurring nearest in time to the index date within a ± 90-day window. An exception was made for laboratory findings with multiple values on the same day in which case the baseline value is defined as the mean of the available measurements. The time in years from 30 days after the date of the second FIB-4 measurement to the date of a liver-related, cardiovascular-related, or any long-term outcome was calculated, excluding patients with cardiovascular events prior to the liver biopsy index date from analysis of both cardiovascular-related events and overall outcomes. For this analysis, the Fine-Gray competing risk model was used to investigate the association of the time to an outcome event with changes in FIB-4, defined as the difference in value at 360 days (±90 days) follow-up versus baseline. Patients with clinical outcome of interest or lost to
follow-up between baseline and 30 days past the date of 360-day FIB-4 follow-up were excluded from the analysis. Crude HRs and adjusted HRs were reported. Covariates included baseline FIB-4 value, baseline age, gender, baseline diabetes, and baseline weight loss surgery. No time-varying variable was used in regression models.

List of ICD and CPT codes

Clinical outcome
codes.xlsx

List of exclusion ICD codes

ICM exclusion codes.xlsx

SDC TABLE 1. Additional baseline characteristics (NAS and components) stratified by baseline stages of fibrosis.

Grade/score	F0–1<br>*n* = 400	F2<br>*n* = 123	F3<br>*n* = 128	F4<br>*n* = 51	Total<br>*N* = 702
**Steatosis, n (%)**					
0	3 (1)	2 (2)	1 (1)	2 (4)	8 (1)
1	184 (46)	47 (38)	55 (43)	28 (55)	314 (45)
2	143 (36)	50 (41)	40 (31)	13 (25)	246 (35)
3	70 (18)	24 (20)	32 (25)	8 (16)	134 (19)
**Lobular inflammation, n (%)**					
0	63 (16)	23 (19)	19 (15)	7 (14)	112 (16)
1	186 (46)	60 (49)	64 (50)	23 (45)	333 (47)
2	37 (9)	11 (9)	14 (11)	4 (8)	66 (9)
3	1 (<1)	0	1 (1)	2 (4)	4 (1)
Not reported	113 (28)	29 (24)	30 (23)	15 (29)	187 (27)
**Hepatocyte ballooning, n (%)**					

0	26 (6)	3 (2)	3 (2)	1 (2)	33 (5)
1	257 (64)	81 (66)	90 (70)	33 (65)	461 (66)
2	9 (2)	3 (2)	8 (6)	3 (6)	23 (3)
Not reported	108 (27)	36 (29)	27 (21)	14 (27)	185 (26)
**NAS total, n (%)**					
1	7 (2)	0	0	0	7 (1)
2	35 (9)	9 (7)	9 (7)	3 (6)	56 (8)
3	68 (17)	23 (19)	21 (16)	8 (16)	120 (17)
4	72 (18)	19 (15)	27 (21)	6 (12)	124 (18)
5	32 (8)	15 (12)	17 (13)	8 (16)	72 (10)
6	10 (2)	2 (2)	4 (3)	1 (2)	17 (2)
Not reported	176 (44)	55 (45)	50 (39)	25 (49)	306 (44)

Abbreviations: F, fibrosis stage; N, number of patients total; n, number of patients with each grade/score; NAS, non-alcoholic fatty liver disease activity score.

SDC TABLE 2. Key baseline characteristics stratified by baseline grades of hepatocyte ballooning.

	HB0	HB1	HB2	Total
	***n* = 33**	***n* = 461**	***n* = 23**	***N* = 702**
**Demographics**				
Female, %	64	71	83	70
Age, median (IQR), years	47 (38–58)	50 (43–58)	53 (44–58)	50 (42–58)
BMI, median (IQR), kg/m^2	44 (38–52)	43 (37–50)	36 (35–43)	44 (37–50)
White, %	88	89	87	90
Hispanic, %	6	2	0	2
Smoker, %	36	37	26	33
Follow-up, median (IQR), years	4.2 (2.5–7.1)	4.6 (2.6–7.2)	4.0 (2.2–7.1)	4.7 (2.8–7.6)
**Medical history**				
Obesity, %	79	81	74	80
Hypertension, %	67	72	52	72
Dyslipidemia, %	48	55	22	54
Type 2 diabetes, %	33	52	70	51
Prior weight-loss surgery, %	82	69	26	68

BMI was evaluated in 685 patients. Smoking status was assessed as ‘ever’, ‘never’, or not reported.

Abbreviations: BMI, body mass index; HB, hepatocyte ballooning grade; IQR, interquartile range.

SDC TABLE 3. Key baseline characteristics stratified by baseline grades of lobular inflammation.

	LI0	LI1	LI2–3	Total
	***n* = 112**	***n* = 333**	***n* = 70**	***N* = 702**
**Demographics**				
Female, %	71	67	77	70
Age, median (IQR), years	49 (42–58)	50 (42–58)	52 (45–59)	50 (42–58)
BMI, median (IQR), kg/m^2	44 (39–50)	43 (36–50)	42 (37–48)	44 (37–50)
White, %	93	89	90	90
Hispanic, %	4	2	1	2
Smoker, %	34	33	36	33
Follow-up, median (IQR), years	4.6 (3.2–7.6)	5.1 (2.8–7.5)	3.9 (1.8–5.6)	4.7 (2.8–7.6)
**Medical history**				
Obesity, %	79	81	80	80
Hypertension, %	72	71	70	72
Dyslipidemia, %	54	54	53	54
Type 2 diabetes, %	53	51	54	51
Prior weight-loss surgery, %	77	66	63	68

BMI was evaluated in 685 patients. Smoking status was assessed as ‘ever’, ‘never’, or not reported.

Abbreviations: BMI, body mass index; IQR, interquartile range; LI, lobular inflammation grade.

SDC TABLE 4. Key baseline characteristics stratified by baseline scores of steatosis.

	S0–1	S2	S3	Total
	***n* = 322**	***n* = 246**	***n* = 134**	***N* = 702**
**Demographics**				
Female, %	67	68	78	70
Age, median (IQR), years	51 (42–59)	49 (41–57)	49 (41–56)	50 (42–58)
BMI, median (IQR), kg/m^2	44 (37–51)	43 (37–49)	43 (37–48)	44 (37–50)
White, %	88	89	96	90
Hispanic, %	2	2	0	2
Smoker, %	32	32	40	33
Follow-up, median (IQR), years	4.5 (2.6–7.3)	4.9 (2.9–7.8)	4.8 (3.0–7.7)	4.7 (2.8–7.6)
**Medical history**				
Obesity, %	81	80	80	80
Hypertension, %	71	73	69	72
Dyslipidemia, %	54	51	57	54
Type 2 diabetes, %	53	48	51	51
Prior weight-loss surgery, %	69	68	68	68

BMI was evaluated in 685 patients. Smoking status was assessed as ‘ever’, ‘never’, or not reported.

Abbreviations: BMI, body mass index; IQR, interquartile range; S, steatosis score.

SDC TABLE 5. Hazard ratios and incidence rates for liver-related outcomes, incident cirrhosis, and cardiovascular-related outcomes, stratified by baseline fibrosis stage (overall and individual events).

	N	Events/person-years at risk	Incidence rate per 100 person-years	HR (95% CI), *p* value
**Liver-related outcomes**				
F4	51	17/174.7	9.73	REF
F3	128	17/612.5	2.78	0.42 (0.21–0.84), $p = 0.015$
F2	123	3/691.2	0.43	0.07 (0.02–0.28), $p \leq 0.001$
F0–1	400	15/2167.6	0.69	0.12 (0.05–0.25), $p \leq 0.001$
**Cirrhosis**				
F3	128	24/549.5	4.37	REF
F2	123	1/701.4	0.14	0.04 (0.01–0.29), $p = 0.002$
F0–1	399	18/2144.3	0.84	0.22 (0.12–0.42), $p < 0.001$
**HCC**				
F4	51	4/213.8	1.87	REF
F3	128	2/654.3	0.31	0.20 (0.02–1.76), $p = 0.146$
F2	123	0/701.6	0.00	NC
F0–1	399	0/2222.6	0.00	NC

	N	Events/person-years at risk	Incidence rate per 100 person-years	HR (95% CI), *p* value
**MELD score ≥15**				
F4	51	11/196.8	5.59	REF
F3	128	6/630.1	0.95	0.39 (0.13–1.21) $p = 0.102$
F2	123	0/701.6	0.00	NC
F0–1	399	0/2222.6	0.00	NC
**Liver transplant**				
F4	51	0/219	0.00	REF
F3	128	0/654.4	0.00	NC
F2	123	0/701.6	0.00	NC
F0–1	400	1/2241	0.04	NC
**Hepatic decompensation**				
F4	51	13/182.9	7.11	REF
F3	128	15/627.9	2.39	0.47 (0.22–1.00), p = 0.05
F2	123	3/691.2	0.43	0.09 (0.02–0.37), $p < 0.001$
F0–1	399	14/2152.2	0.65	0.14 (0.06–0.31), $p < 0.001$
**Cardiovascular-related outcomes**				

	N	Events/person-years at risk	Incidence rate per 100 person-years	HR (95% CI), *p* value
F4	47	6/178.3	3.37	REF
F3	119	10/571.7	1.75	0.46 (0.15–1.39), $p = 0.169$
F2	113	7/564.3	1.24	0.41 (0.12–1.37), $p = 0.148$
F0–1	381	18/2039.4	0.88	0.24 (0.08–0.70), $p = 0.009$
**Heart failure**				
F4	47	5/183.9	2.72	REF
F3	119	4/589.2	0.68	0.16 (0.04–0.70), $p = 0.015$
F2	113	6/581.6	1.03	0.26 (0.07–1.01), $p = 0.052$
F0–1	381	8/2066.2	0.39	0.08 (0.02–0.30), $p < 0.001$
**Myocardial infarction**				
F4	47	3/188.4	1.59	REF
F3	119	0/600.3	0.00	NC
F2	113	1/582.4	0.17	0.13 (0.01–1.79), $p = 0.128$
F0–1	381	2/2074.2	0.10	0.06 (0.00–0.69), $p = 0.024$
**Ischemic stroke**				
F4	47	2/200.9	1.00	REF

	N	Events/person-years at risk	Incidence rate per 100 person-years	HR (95% CI), *p* value
F3	119	6/587.8	1.02	1.29 (0.30–5.59), *p* = 0.729
F2	113	2/591.7	0.34	0.52 (0.06–4.97), *p* = 0.574
F0–1	381	7/2072.1	0.34	0.52 (0.10–2.72), *p* = 0.439
**Unstable angina**				
F4	47	0/203.6	0.00	REF
F3	119	2/589.6	0.34	NC
F2	113	1/592.8	0.17	NC
F0–1	381	4/2068.3	0.19	NC
**Coronary revascularization**				
F4	47	1/200.5	0.50	REF
F3	119	0/600.3	0.00	NC
F2	113	0/598.5	0.00	NC
F0–1	381	3/2082.5	0.14	0.23 (0.05–0.98), *p* = 0.046
**Any long-term outcome**				
F4	47	16/149.7	10.69	REF
F3	119	25/534.4	4.68	0.52 (0.26–1.02), *p* = 0.057

	N	Events/person-years at risk	Incidence rate per 100 person-years	HR (95% CI), *p* value
F2	113	10/554	1.81	0.23 (0.10–0.54), $p < 0.001$
F0–1	381	27/1988.9	1.36	0.16 (0.08–0.33), $p < 0.001$

N refers to total number of patients at risk. All HRs were adjusted for sex, baseline age, diabetes, and weight loss surgery status, unless otherwise specified.

Liver-related outcomes were defined as any of the following: HCC, MELD ≥15, hepatic decompensation, liver transplant, or liver-related mortality. Cirrhosis was analysed separately in patients with F0–3 at baseline and not included in liver-related outcomes. Cardiovascular-related outcomes included any of the following: heart failure, myocardial infarction, ischemic stroke, unstable angina, and coronary revascularization, and cardiovascular-related mortality for patients with no cardiovascular outcome events at baseline. Any long-term outcome was defined as any liver-related [excluding cirrhosis], and cardiovascular-related outcomes, and mortality due to liver, cardiovascular or cancer events.

Abbreviations: CI, confidence interval; F, fibrosis stage; HCC, hepatocellular carcinoma; HR, hazard ratio; MELD, model for end-stage liver disease; NC, not calculated; REF, reference comparator.

SDC TABLE 6. Hazard ratios and incidence rates for liver-related outcomes, incident cirrhosis, and cardiovascular-related outcomes, stratified by baseline hepatocyte ballooning grade (overall and individual events).

	N	**Events/person-years at risk**	**Incidence rate per 100 person-years**	**HR (95% CI), *p* value**	**Multiple imputation, HR (95% CI), *p* value**
**Liver-related outcomes**					
HB2	23	1/112.9	0.89	REF	REF
HB1	461	38/2170.5	1.75	3.01 (0.40–22.46), $p = 0.283$	2.07 (0.47–9.06), $p = 0.332$
HB0	33	1/151.2	0.66	1.56 (0.09–26.14), $p = 0.756$	0.95 (0.08–11.13), $p = 0.965$
**Cirrhosis**					
HB2	20	7/80.9	8.65	REF	REF
HB1	428	27/2048.5	1.32	0.20 (0.08–0.50), $p < 0.001$	0.32 (0.12–0.86), $p = 0.025$
HB0	32	1/149.3	0.67	0.13 (0.02–1.18), $p = 0.071$	0.20 (0.02–1.78), $p = 0.151$
**HCC**					
HB2	23	0/114.7	0.00	REF	REF
HB1	461	5/2277.8	0.22	NC	NC
HB0	33	0/153.1	0.00	NC	NC
**MELD score ≥15**					

	N	Events/person-years at risk	Incidence rate per 100 person-years	HR (95% CI), *p* value	Multiple imputation, HR (95% CI), *p* value
HB2	23	0/114.7	0.00	REF	REF
HB1	461	12/2253.8	0.53	NC	NC
HB0	33	0/153.1	0.00	NC	NC
**Liver transplant**					
HB2	23	0/114.7	0.00	REF	REF
HB1	461	0/2282.1	0.00	NC	NC
HB0	33	0/153.1	0.00	NC	NC
**Hepatic decompensation**					
HB2	23	1/112.9	0.89	REF	REF
HB1	461	35/2183.7	1.60	2.83 (0.38–21.17), $p = 0.311$	2.93 (0.35–24.64), $p = 0.323$
HB0	33	1/151.2	0.66	1.62 (0.10–27.15), $p = 0.737$	1.72 (0.11–26.89), $p = 0.700$
**Cardiovascular-related outcomes**					
HB2	22	2/107.6	1.86	REF	REF
HB1	433	20/2049.1	0.98	0.81 (0.17–3.94), $p = 0.797$	0.99 (0.26–3.73), $p =0.991$
HB0	32	0/146	0.00	NC	NC

	N	Events/person-years at risk	Incidence rate per 100 person-years	HR (95% CI), *p* value	Multiple imputation, HR (95% CI), *p* value
**Heart failure**					
HB2	22	0/110.9	0.00	REF	REF
HB1	433	14/2060.8	0.68	NC	NC
HB0	32	0/146	0.00	NC	NC
**Myocardial infarction**					
HB2	22	0/110.9	0.00	REF	REF
HB1	433	3/2074.4	0.14	NC	NC
HB0	32	0/146	0.00	NC	NC
**Ischemic stroke**					
HB2	22	2/107.6	1.86	REF	REF
HB1	433	6/2087.6	0.29	0.36 (0.06–2.33), $p = 0.283$	0.52 (0.07–3.88), $p = 0.520$
HB0	32	0/146	0.00	NC	0.00 (0.00–4.58), $p = 0.077$
**Unstable angina**					
HB2	22	0/110.9	0.00	REF	REF
HB1	433	2/2080.6	0.10	NC	NC

	N	Events/person-years at risk	Incidence rate per 100 person-years	HR (95% CI), *p* value	Multiple imputation, HR (95% CI), *p* value
HB0	32	0/146	0.00	NC	NC
**Coronary revascularization**					
HB2	22	0/110.9	0.00	REF	REF
HB1	433	3/2090.5	0.14	NC	NC
HB0	32	0/146	0.00	NC	1.16 (0.29–4.72), *p* = 0.835
**Any long-term outcome**					
HB2	22	2/107.6	1.86	REF	REF
HB1	433	48/1961.5	2.45	1.82 (0.43–7.71), *p* = 0.418	1.88 (0.55–6.41), *p* = 0.315
HB0	32	1/144.1	0.69	0.66 (0.06–7.56). *p* = 0.74	1.40 (0.13–15.26), *p* = 0.783

N refers to total patients at risk. All HRs were adjusted for sex, baseline age, diabetes, and weight loss surgery status, unless otherwise specified.

Liver-related outcomes were defined as any of the following: HCC, MELD ≥15, hepatic decompensation, liver transplant, or liver-related mortality. Cirrhosis was analysed separately in patients with F0–3 at baseline and not included in liver-related outcomes. Cardiovascular-related outcomes included any of the following: heart failure, myocardial infarction, ischemic stroke, unstable angina, and coronary revascularization, and cardiovascular-related mortality for patients with no cardiovascular outcome events at baseline. Any long-term outcome was defined as any liver-related [excluding cirrhosis], and cardiovascular-related outcomes, and mortality due to liver, cardiovascular or cancer events.

Abbreviations: CI, confidence interval; HB, hepatocyte ballooning grade; HCC, hepatocellular carcinoma; HR, hazard ratio; MELD, model for end-stage liver disease; NC, not calculated; REF, reference comparator.

SDC TABLE 7. Hazard ratios and incidence rates for liver-related outcomes, incident cirrhosis, and cardiovascular-related outcomes, stratified by baseline lobular inflammation grade (overall and individual events).

	N	Events/person-years at risk	Incidence rate per 100 person-years	HR (95% CI), *p* value	Multiple imputation, HR (95% CI), *p* value
**Liver-related outcomes**					
LI2–3	70	9/287	3.14	REF	REF
LI1	333	21/1747.5	1.20	0.46 (0.20–1.07), $p = 0.072$	0.53 (0.20–1.37), $p = 0.190$
LI0	112	7/634.6	1.10	0.45 (0.16–1.29), $p = 0.139$	0.49 (0.16–1.52), $p = 0.217$
**Cirrhosis**					
LI2–3	64	8/261.2	3.06	REF	REF
LI1	310	21/1639.4	1.28	0.42 (0.19–0.97), $p = 0.041$	0.44 (0.20–1.00), $p = 0.050$
LI0	105	10/590	1.69	0.58 (0.22–1.50), $p = 0.258$	0.66 (0.26–1.68), $p = 0.380$
**HCC**					
LI2–3	70	0/318.4	0.00	REF	REF
LI1	333	4/1805.6	0.22	NC	0.53 (0.07–4.24), $p = 0.550$
LI0	112	0/671.2	0.00	NC	0.00 (0.00–13.06), $p = 0.093$
**MELD score ≥15**					

	N	Events/person-years at risk	Incidence rate per 100 person-years	HR (95% CI), *p* value	Multiple imputation, HR (95% CI), *p* value
LI2–3	70	2/314	0.64	REF	REF
LI1	333	6/1794.8	0.33	0.42 (0.08–2.20), *p* = 0.302	0.51 (0.11–2.43), *p* = 0.400
LI0	112	3/653.2	0.46	0.72 (0.12–4.50), *p* = 0.727	1.18 (0.17–8.35), *p* = 0.867
**Liver transplant**					
LI2–3	70	0/318.4	0.00	REF	REF
LI1	333	0/1810.3	0.00	NC	NC
LI0	112	0/671.2	0.00	NC	NC
**Decompensation**					
LI2–3	70	9/287	3.14	REF	REF
LI1	333	16/1763.4	0.91	0.34 (0.14–0.80), *p* = 0.013	0.31 (0.14–0.66), *p* = 0.003
LI0	112	7/639.4	1.09	0.44 (0.16–1.24), *p* = 0.121	0.39 (0.14–1.10), *p* = 0.076
**Cardiovascular-related outcomes**					
LI2–3	65	4/277.3	1.44	REF	REF
LI1	314	19/1626.1	1.17	0.55 (0.18–1.68), *p* = 0.295	0.70 (0.32–1.52) *p* = 0.364
LI0	104	11/535.7	2.05	1.29 (0.40–4.18) *p* =0.669	1.10 (0.45–2.70), *p* = 0.835

	N	Events/person-years at risk	Incidence rate per 100 person-years	HR (95% CI), *p* value	Multiple imputation, HR (95% CI), *p* value
**Heart failure**					
LI2–3	65	0/290.9	0.00	REF	REF
LI1	314	13/1647.7	0.79	NC	1.01 (0.31–3.29), *p* = 0.980
LI0	104	6/558.6	1.07	NC	1.34 (0.36–4.99), *p* = 0.659
**Myocardial infarction**					
LI2–3	65	0/290.9	0.00	REF	REF
LI1	314	2/1665.2	0.12	NC	0.33 (0.07–1.53), *p* = 0.156
LI0	104	3/563.2	0.53	NC	0.57 (0.13–2.53), *p* = 0.456
**Ischemic stroke**					
LI2–3	65	4/277.3	1.44	REF	REF
LI1	314	6/1662.2	0.36	0.13 (0.03–0.50), *p* = 0.003	0.21 (0.08–0.56), *p* = 0.002
LI0	104	4/588.3	0.68	0.25 (0.06–1.08), *p* = 0.062	0.40 (0.14–1.15), *p* = 0.088
**Unstable angina**					
LI2–3	65	0/290.9	0.00	REF	REF
LI1	314	3/1665.4	0.18	NC	0.18 (0.03–1.03), *p* = 0.054

	N	Events/person-years at risk	Incidence rate per 100 person-years	HR (95% CI), *p* value	Multiple imputation, HR (95% CI), *p* value
LI0	104	3/571.6	0.52	NC	0.44 (0.07–2.68), *p* = 0.374
**Coronary revascularization**					
LI2–3	65	0/290.9	0.00	REF	REF
LI1	314	1/1679.7	0.06	NC	0.08 (0.02–0.34), *p* < 0.001
LI0	104	2/583.8	0.34	NC	0.22 (0.05–0.96), *p* = 0.043
**Any long-term outcome**					
LI2–3	65	11/257.9	4.26	REF	REF
LI1	314	31/1586.8	1.95	0.44 (0.22–0.89), *p* = 0.022	0.58 (0.34–1.00), *p* = 0.050
LI0	104	17/504.6	3.37	0.85 (0.39–1.84), *p* = 0.679	0.80 (0.41–1.57), *p* = 0.513

N refers to total patients at risk. All HRs were adjusted for sex, baseline age, diabetes, and weight loss surgery status, unless otherwise specified.

Liver-related outcomes were defined as any of the following: HCC, MELD ≥15, hepatic decompensation, liver transplant, or liver-related mortality. Cirrhosis was analysed separately in patients with F0–3 at baseline and not included in liver-related outcomes. Cardiovascular-related outcomes included any of the following: heart failure, myocardial infarction, ischemic stroke, unstable angina, and coronary revascularization, and cardiovascular-related mortality for patients with no cardiovascular outcome events at baseline. Any long-term outcome was defined as any liver-related [excluding cirrhosis], and cardiovascular-related outcomes, and mortality due to liver, cardiovascular or cancer events.

Abbreviations: CI, confidence interval; HCC, hepatocellular carcinoma; HR, hazard ratio; LI, lobular inflammation; MELD, model for end-stage liver disease; NC, not calculated; REF, reference comparator.

SDC TABLE 8. Exploratory analysis of FIB-4: adjusted hazard ratios for liver-related outcomes.

	FIB-4	
	**HR (95% CI)**	***p* value**
**360-day change in FIB-4 (n = 268)**	2.04 (0.99–4.19)	0.053
**Baseline FIB-4**	1.57 (1.00–2.46)	0.048
**Baseline age**	0.98 (0.93–1.04)	0.508
**Sex (male vs. female]**	1.33 (0.47–3.78)	0.597
**Baseline diabetes (yes vs. no)**	1.57 (0.56–4.38)	0.388
**Baseline weight-loss surgery (yes vs. no)**	0.41 (0.13–1.28)	0.124

Follow-up value extracted 360 days (±90 days) from the patient's index date (date of the first MASH liver biopsy). Patients with liver-related event before the second FIB-4 measurement date +30 days were excluded.

Abbreviations: CI, confidence interval; FIB-4, fibrosis-4 index; HR, hazard ratio.
